# Polaritonic Control of Blackbody Infrared Radiative Dissociation

**DOI:** 10.1021/acs.jpclett.5c01475

**Published:** 2025-07-18

**Authors:** Enes Suyabatmaz, Gustavo J. R. Aroeira, Raphael F. Ribeiro

**Affiliations:** † Department of Physics, 1371 Emory University, Atlanta, Georgia 30322, United States; ‡ Department of Chemistry and Cherry Emerson Center for Scientific Computation, Emory University, Atlanta, Georgia 30322, United States

## Abstract

Vibrational strong light–matter coupling offers a promising approach for controlling chemical reactivity with infrared microcavities. While recent research has examined potential mechanisms for this phenomenon, many important questions remain, including what type of reactions can be modified and to what extent this modification can be achieved. In this study, we explore the dynamics of Blackbody Infrared Radiative Dissociation (BIRD) in microcavities under weak and strong light–matter interaction regimes. Using a Master equation approach, we simulate the effects of infrared field confinement and polariton formation on BIRD rates for diatomic molecules weakly coupled to the radiation field. We present a framework explaining how infrared microcavities influence BIRD kinetics, highlighting the importance of overtone transitions in the process. Our findings outline conditions under which significant enhancement or mild suppression of BIRD rates can be achieved, offering insights into practical limitations and new strategies for controlling chemistry within infrared resonators.

## Graphical abstract

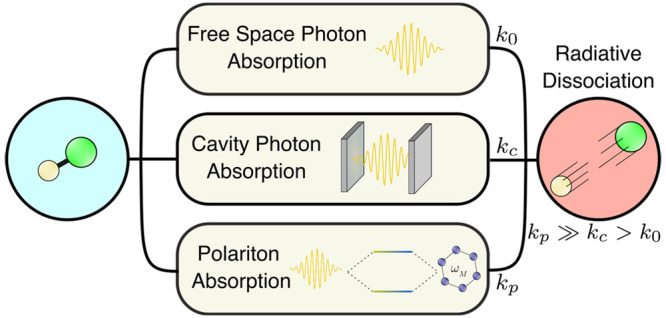

## 1 Introduction

Chemical reaction control is critical in applications ranging from industrial catalysis to medicine. Conventional methods for manipulating reaction rates involve varying the temperature, pressure, or catalyst composition. An unconventional approach for controlling thermal reaction kinetics has been recently reported^1–4^ employing infrared (IR) microcavities^5^ consisting of two moderate-quality mirrors separated by a distance *L*_C_ of the order of IR wavelengths resonant with typical molecular vibrations.^6^ When a collection of molecules with sufficiently large IR oscillator strength is embedded in a resonant microcavity, vibrational strong coupling occurs as signaled by the emergence of hybrid quasiparticles (vibrational polaritons) consisting of a superposition of molecular and electromagnetic modes of the confined device.^7–9^ Recent theoretical research has probed potential mechanisms for the observed polariton effects on chemical reaction rates,^4,10–19^ and experimental progress toward the characterization of gas-phase reactivity under strong light–matter coupling has also been reported.^20,21^ Still, several fundamental questions remain,^22,23^ including what types of reactions can be controlled with an optical microcavity and to what extent this control can be exerted. In this study, we investigate gas-phase unimolecular dissociation activated by the absorption of microcavity blackbody infrared radiation^24^ under weak and strong coupling with a suitable material.

Blackbody infrared radiative dissociation (BIRD) was proposed by Perrin in 1913 as a mechanism for gas phase unimolecular dissociation.^25^ However, experimental observations in the 1920s ruled BIRD out in favor of the collisional activation mechanism.^26–28^ Several decades later, the BIRD hypothesis was proven appropriate at sufficiently low pressures.^29–31^ Since then, BIRD has become a valuable tool for investigating the thermal dissociation kinetics of molecular ions and clusters and has been observed in several systems, including ion clusters, transition–metal complexes, and biopolymers.^24^

The thermal radiation spectrum heavily influences the BIRD rates. Given that free space and microcavity electromagnetic density of states and thermal radiation density can be significantly different,^5^ microcavity BIRD is expected to proceed with different rates compared to free space BIRD. However, the extent of this change, whether suppression or enhancement can be achieved, and if strong light–matter coupling introduces new possibilities relative to the weak coupling regime remain open questions. In this study, we address these questions by examining the dissociation of diatomic molecules driven by multiphoton or multipolariton absorption in microcavities. The qualitative conclusions drawn from this analysis are expected to be generalizable to polyatomic systems.

The primary aim of this work is to characterize thermal radiative dissociation in microcavities. Whereas prior studies employed hierarchical equations of motion and analytical rate theories based on Fermi’s golden rule to explore energy-diffusion-limited reactivity modeled by double-well potentials,^14,16,17,32–34^ here we employ a Pauli master equation to investigate irreversible bond breaking with a Morse potential in a scenario where the microcavity or polaritons act solely as passive modifiers of the electromagnetic environment. Our methodology further differs by its microscopically detailed treatment of mechanical and electrical anharmonicity inherent to reactivity processes and by its inclusion of the coupling of both fundamental and overtone vibrational transitions to the modified photon density of states, thereby providing a detailed description of the microcavity effect on the multiple reactive pathways available in BIRD.

We employ a Pauli Master equation to investigate BIRD (i) in free space, (ii) under weak coupling with a nearly empty microcavity, and (iii) under weak coupling with a polaritonic material. Our results give upper bounds for BIRD enhancement and suppression in weakly and strongly coupled microcavities with perfect mirrors (see SI Sec. 8 for a discussion of BIRD rates in leaky microcavities) and identify overtone transitions as crucial ingredients for the observed modulation of BIRD. In Sec. [Sec sec2], we describe our methodology, Sec. [Sec sec3] provides the main results and related discussion, and Sec. [Sec sec4] summarizes our findings and conclusions.

## 2 Computational Methods

### 2.1 Pauli Master Equation for Diatomic BIRD

Our analysis of BIRD in microcavities employs the same kinetic framework that has proven successful for BIRD studies in free space.^24,30,35,36^ Specifically, we propagate the populations of bound vibrational levels with a Pauli master equation^37^ including transition rates obtained from Fermi’s golden rule.^38^ This choice is justified because the infrared absorption and emission events of interest are orders of magnitude slower than rovibrational decoherence, which randomizes vibrational coherences on a much faster time scale. The resulting separation of time scales renders the diatomic reduced density matrix effectively diagonal (in the Morse Hamiltonian eigenstate basis) well before any population transfer occurs, allowing the Liouville–von Neumann equation to be coarse-grained to the Pauli form.^37,39,40^

We neglect collisional activation and assume that dissociation is driven exclusively by photon absorption, consistent with the conditions of BIRD experiments.^24,30^ In such experiments, the use of dilute gas samples at low pressure ensures that intermolecular collisions are negligible. External fields are applied to trap and stabilize molecular trajectories within the interior of the reaction vessel. This prevents molecules from exchanging energy with walls and allows radiative processes to dominate.^41–43^ Rotational degrees of freedom are ignored, since they are weakly coupled to vibrational transitions, and the rotational dynamics are essentially classical at the temperatures of interest to BIRD. We also neglect Doppler and lifetime broadening throughout. For the sake of simplicity, we assumed that the thermalization of the background radiation is faster than all of the considered radiative transitions. We analyzed microcavity effects on BIRD that occurs via two distinct mechanisms. In the first, denoted dissociation via the doorway state, the dissociation proceeds only via the highest-energy bound state (the doorway state). In this case, the rate of change of the population in the *i*th vibrational state (*N*_*i*_) is given by

1 $$\frac{dN_{i}\left(t\right)}{dt}=\overset{i_{\max}}{\underset{j\neq i}{\sum }}\left[k_{ij}N_{j}\left(t\right)-k_{ji}N_{i}\left(t\right)\right]-k_{\mathrm{loss}}N_{i}\left(t\right)\delta_{i,i_{\max}}$$

where *k*_*ij*_ denotes the rate of absorption when *i* > *j* and emission when *i* < *j* and *i*_max_ is the highest energy bound state (Figure 1a). As mentioned, this state is only loosely bound and is assumed to undergo irreversible dissociation at a rate *k*_loss_ ≫ *k*_*ij*_ ∀ *i*, *j*. Eq 1 can be written in matrix notation as

2 $$\frac{d\left[N\right]}{dt}=-J\cdot \left[N\right]$$

where **N** is a column vector containing the vibrational populations and **J** is the transition matrix. The lowest eigenvalue λ_0_ of **J** is the rate constant of the unimolecular reaction^44,45^ of the described mechanism. Note that this mechanism does not consider dissociation from intermediate vibrational levels (*i* < *i*_max_). Thus, our results are expected to be valid for BIRD promoted by multiphoton dissociation through a particular doorway state, which in our case is the highest-energy bound state.

**Figure 1:**
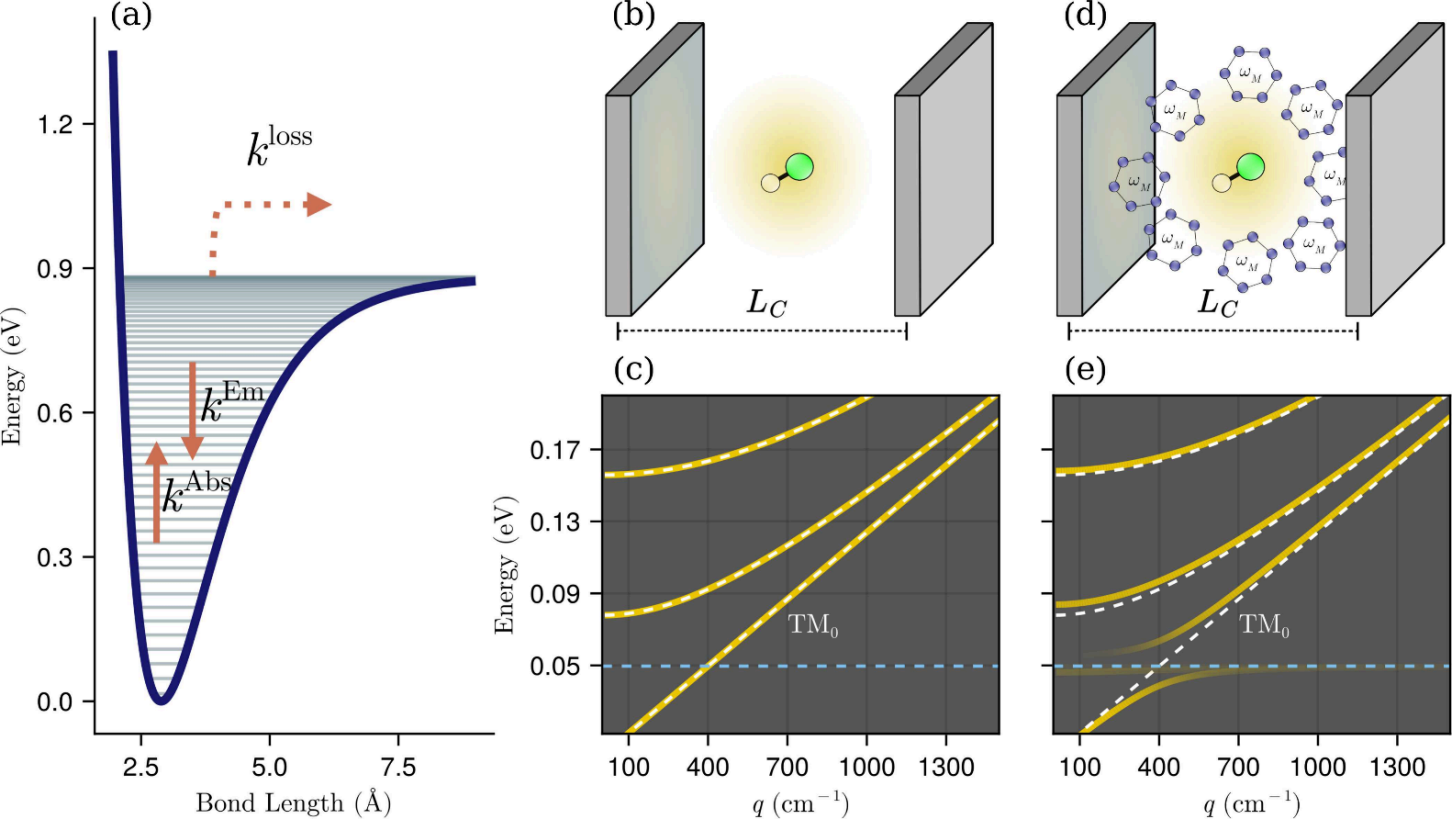
(a) Illustration of the Morse potential describing the vibrational states and considered transitions of the NaLi molecule. (b) Illustration of a diatomic molecule inside a high quality planar microcavity with longitudinal length *L*_C_. (c) Dispersion curves of transverse-electric (TE) and transverse-magnetic (TM) microcavity modes. (d) Scheme illustrating a reactive diatomic molecule inside a planar resonator strongly coupled to a host material system with significant oscillator strength and transition frequency ω_M_ (dashed line at ω = 0.05 eV). In this setup, the diatomic dissociation is mediated by absorption of thermal polaritons. (e) Illustration of polariton dispersion curves.

The second dissociation mechanism considered allows for direct radiative dissociation from any bound state of the diatomic molecule. Compared to eq 1, this introduces a dissociation channel for each bound state, characterized by a loss rate of *k*_loss_^*i*^. The corresponding population rate equations are given by

$$\frac{dN_{i}\left(t\right)}{dt}=\overset{i_{\max}}{\underset{j\neq i}{\sum }}\left[k_{ij}N_{j}\left(t\right)-k_{ji}N_{i}\left(t\right)\right]-k_{\mathrm{loss}}^{i}N_{i}\left(t\right)$$ 3

The transition matrix **J** now includes contributions from bound-continuum decay rates in all diagonal elements. The presence of *k*_loss_^*i*^ for all *i* values ensures that the vibrational population can be irreversibly removed from each level via dissociation. The smallest eigenvalue λ_0_ of **J** continues to govern the long-term dynamics of the system.

### 2.2 Radiative Transition Rates

Assuming the molecular system that undergoes BIRD is weakly coupled to the radiation field, the transition rates *k*_*ij*_ can be obtained from perturbation theory (Fermi’s golden rule^46,47^ ) and written in terms of Einstein coefficients in free space^48,49^ and their generalizations in a weakly coupled and polaritonic microcavity. Rotational dynamics is much faster than the thermal infrared radiative transition, so we employ isotropically averaged radiative rates in the long-wavelength limit (electrical dipole approximation) throughout this work.

In free space, the spontaneous emission rate is given by the Einstein *A* coefficient.^48,49^ In the case where spontaneous emission induces the *i* → *j* transition with *i* > *j* this coefficient is given by

4 $$k_{ij}^{\mathrm{SpEm}}=A_{ij}=\frac{\omega_{ij}{\left|\mu_{ij}\right|}^{2}\pi }{3\varepsilon_{0}\hbar }D_{0}\left(\omega_{ij}\right)$$

where ε_0_ and ℏ are the vacuum electrical permittivity and reduced Plank’s constant, respectively, ω_*ij*_ = (*E*_*i*_ – *E*_*j*_)/ℏ is the transition frequency, μ_*ij*_ is the corresponding transition dipole moment, and *D*_0_(ω) = ω^2^/π^2^ *c* ^3^ is the free-space radiative density of states (DOS). The Einstein *B* coefficient associated with stimulated emission and absorption processes involving states *i* and *j* is given by *B*_*ij*_ = π|μ_*ij*_|^2^/(3ε_0_ℏ^2^). The corresponding thermal stimulated emission and absorption rate constants are given by

5 $$k_{ij}^{\mathrm{Abs}}\left(T\right)=k_{ij}^{\mathrm{StEm}}\left(T\right)=B_{ij}\rho_{0}\left(\omega_{ij},T\right)$$

where ρ_0_(ω, *T*) is the free space blackbody radiation energy density

6 $$\rho_{0}\left(\omega ,T\right)=\hbar \omega n_{\mathrm{BE}}\left(\omega ,T\right)D_{0}\left(\omega \right)$$

and *n*_BE_(ω, *T*) is the Bose–Einstein distribution thermal occupation number *n*_BE_(ω, *T*) = 1/[exp­(ℏω/*k*_B_ *T*) – 1][Bibr ref50] and *k*_B_ is the Boltzmann constant.

The final pieces necessary to compute the rate constants are the transition dipole matrix μ_*ij*_ and the frequencies ω_*ij*_. We estimate the latter assuming the electronic ground-state potential energy curve of the examined diatomic is given by a Morse potential (Figure 1a).^51^ The bound wave functions associated with this Morse potential are employed to compute the vibrational transition dipole moments μ_*ij*_ according to

7 $$\mu_{ij}=\int \psi_{i}^{*}\left(r\right)\mu \left(r\right)\psi_{j}\left(r\right)\;dr$$

where ψ_*i*_(*r*) represents the *i*th vibrational level wave function and the dipole function μ­(*r*) models the nonlinear change in the (electronic ground-state) molecular electrical dipole with the bond length *r*.^52^

The rates *k*_loss_^*i*^ for radiative absorption into the continuum by each bound state *i* are obtained by a simple generalization of eq 5 as discussed in the Supporting Information.

We investigated the thermal IR radiative dissociation of the NaLi molecule. The electrical dipole function and Morse potential were obtained via interpolation from previous CCSDT/cc-pCVQZ calculations available in the literature^53^ (see Supporting Information for further details). Morse potential parameters are listed in Table 1. The transition rates *k*_*ij*_ are proportional to the electromagnetic density of states *D*(ω), suggesting that BIRD rates can be controlled by placing the reactive molecule inside a microcavity. To examine this scenario, we adapt the free space formalism described above to arbitrary photonic devices by replacing *D*_0_(ω) in eqs 4 and 6 with the appropriate DOS for a weakly coupled microcavity *D*_C_(ω).

**Table 1:** Parameters Characterizing the Morse Potential Associated with NaLi

Parameter	Notation (unit)	Value
Electronic ground-state dissociation energy	*D*_e_ (eV)	0.882
Equilibrium bond length	*r*_e_ (Å)	2.895
Harmonic frequency	ω_e_ (cm^–1^)	257.4
Anharmonicity	ω_e_χ_e_ (cm^–1^)	2.33
Reduced mass	*m*_r_ (au)	5.331

In a planar empty Fabry–Perot microcavity composed of two parallel mirrors (with unit reflectivity for simplicity) separated by a distance *L*_C_, (Figure 1b), the frequencies of the electromagnetic modes are given by

8 $$\omega_{C}\left(m,q\right)=c\sqrt{q^{2}+\frac{m^{2}\pi^{2}}{{L_{C}}^{2}}}$$

where $q=\left(q_{x},q_{y}\right)\in R^{2}$ is the in-plane projection of the mode wave vector, *q* ^2^ = *q*_*x*_^2^ + *q*_*y*_^2^, and *m* is an integer quantum number that identifies the longitudinal wave vector *k*_*z*_ = *m*π/*L*_C_ and thus the photonic energy bands in Figure 1c. For transverse-magnetic (TM) modes *m* = 0, 1, 2, etc. whereas *m* = 1, 2, 3, etc. for transverse-electric (TE) modes.^54,55^ Including the TM_0_ mode here is crucial, as otherwise transitions with frequency below the cavity cutoff [ω_C_ (1, 0)] would be impossible.

Using eq 8, it can be shown (see Supporting Information) that the spatially averaged electromagnetic DOS inside a microcavity in the weak coupling regime can be expressed by^56^

9 $$D_{C}\left(\omega \right)=\frac{\omega }{\pi c^{2}L_{C}}\left\lfloor \frac{\omega L_{C}}{\pi c}\right\rfloor +\frac{\omega }{2\pi c^{2}L_{C}}$$

where ⌊*x*⌋ denotes the floor function. This work focuses on establishing an upper limit for microcavity effects on BIRD by assuming perfectly reflecting mirrors. Real microcavities are leaky, so to assess the robustness of our predictions, we also computed the photon density of states and corresponding quantum state transition rates in imperfect microcavities with finite reflectivity and absorbing metallic mirrors using the electromagnetic field dyadic Green function^56^ (see Supporting Information for details).

Lastly, we considered the BIRD scenario in which the diatomic interaction weakly interacts with the electromagnetic component of polariton modes emergent from vibrational strong coupling between the microcavity modes and a material system (solid-state or molecular ensemble) with sufficiently large IR oscillator strength. We emphasize that the polariton modes arise from the collective strong coupling between the cavity and a host material with substantial infrared oscillator strength. The reactive molecule experiences modified radiative dynamics due to changes in the photon density of states induced by the formation of polaritons, as illustrated in Figure 1d,e. Consequently, in this study, the interaction strength between our reactive diatomic and the radiation field is obtained from first-principles without any scaling parameters.

While this polariton-assisted scenario poses challenges for experimental realization, as BIRD generally requires low-density, collision-free conditions, it is introduced here as a theoretical construct that isolates the effect of polariton-modified photonic environments on molecular infrared radiative dissociation. Nonetheless, one could envision suppressing collisions by confining reactive molecules using transparent partitions or external fields, while maintaining exposure to the confined electromagnetic modes.^41–43^ A detailed treatment of these experimental considerations, as well as the role of direct interactions between host and reactive molecules, lies beyond the scope of the present work and warrants future investigation. The formalism discussed above for BIRD in a weakly coupled microcavity can be straightforwardly adapted to model polariton-mediated BIRD (see SI) by replacing the photon density of states *D*_0_(ω) with the *photon-weighted polariton density of states D*_P_(ω).

The Hamiltonian of the strongly coupled molecular subsystem and the microcavity is given in the Power–Zienau–Woolley (dipole) gauge^57,58^

10 $$H=H_{\mathrm{SM}}+H_{L}+H_{\mathrm{INT}}$$

where the interaction *H*_INT_ is given by

11 $$H_{\mathrm{INT}}=\frac{1}{\varepsilon_{0}}\int \left[-D\left(r\right)\cdot P\left(r\right)+\frac{1}{2}P^{2}\left(r\right)\right]\;d^{3}r$$

where **P**(**r**) is the matter polarization density and **D**(**r**) is the electrical displacement field.^59,60^ The pure matter and bare electromagnetic Hamiltonians *H*_SM_ and *H*_L_ generate the dynamics of a collection of free harmonic oscillators corresponding to isotropic uniformly distributed matter vibrations (e.g., from a dispersionless paraelectric material^61,62^ ) and planar microcavity modes with frequency-momentum dispersion given by eq 8. Assuming a strongly coupled 3D isotropic material system with uniform spatial distribution, it follows in the mean-field limit using the standard Bogoliubov transformations^63–65^ that the photon-weighted polariton density of states *D*_P_(ω) can be written as (see SI)­

12 $$D_{P}\left(\omega \right)=\overset{\infty }{\underset{m=0}{\sum }}\left(1-\frac{\delta_{0,m}}{2}\right)\frac{q\left(m,\omega \right)P_{C}\left(\omega \right)}{\pi v_{g}\left(\omega ,q\right)L_{C}}\theta \left[\omega_{C}\left(\omega \right)-m\pi c/L_{C}\right]$$

where θ­(*x*) = 1 for *x* ≥ 0 and vanishes elsewhere, ω_C_(ω) is the frequency of the photon mode that under strong coupling leads to the formation of a polariton with frequency ω, *P*_C_(ω) is the photon content of the polariton modes with frequency ω

13 $$P_{C}\left(\omega \right)=\frac{{\left(\omega^{2}-{\omega_{M}}^{2}-{\Omega_{R}}^{2}\right)}^{2}}{{\left(\omega^{2}-{\omega_{M}}^{2}-{\Omega_{R}}^{2}\right)}^{2}+{\omega_{C}}^{2}\left(\omega \right){\Omega_{R}}^{2}}$$

ω_M_ is the matter oscillator frequency, Ω_R_ is the collective light–matter interaction strength, and the polariton group velocity (*v*_g_) is given by

14 $$v_{g}\left(\omega ,q\right)=\frac{qc^{2}}{2\omega }\left(1\pm \frac{{\omega_{C}}^{2}-{\omega_{M}}^{2}+{\Omega_{R}}^{2}}{\sqrt{{\left({\omega_{C}}^{2}+{\omega_{M}}^{2}+{\Omega_{R}}^{2}\right)}^{2}-4{\omega_{C}}^{2}{\omega_{M}}^{2}}}\right)$$

where the positive sign applies when ω belongs to an upper polariton branch, i.e., ω > ω_C_, and the negative sign is used if ω < ω_C_. In this expression, we omitted the frequency dependence of ω_C_ for simplicity.

This model shows singular behavior for *D*_P_(ω) as ω → ω_M_. In this limit, both the photon content *P*_C_(ω) and the group velocity *v*_g_(ω, *q*) approach zero, but the ratio *P*_C_(ω)/*v*_g_(ω, *q*) → *∞* resulting in *D*_P_(ω) diverging to positive infinity. This singularity is due to the formation of the well-known stopgap region^66–69^ where *D*_P_(ω) = 0. As previous work shows,^70^ introducing an ultraviolet cutoff and material disorder eliminates the singularity of *D*_P_(ω) at ω_M_. Nevertheless, there remains significant enhancement of *D*_P_(ω) in the neighborhood of ω_M_, which depends in a nonuniversal way on disorder and coherence loss mechanisms. Similarly, the stopgap is known to contain weakly coupled states in disordered systems, causing *D*_P_(ω) to become nonzero in this region. However, the photon density of states remains lower in the stopgap in comparison to the weak coupling regime. The robustness of the enhanced and reduced photon-weighted polariton density of states at frequencies ω approaching ω_M_ from the left and right, respectively, supports our choice to use the formally exact expression presented in eq 12 and interpret our results as upper bounds on the effect of polaritons on BIRD in perfect microcavities. The relevance of these bounds to experiments will be shown to depend on the collective light–matter interaction strength and the detuning between transitions relevant to BIRD and ω_M_ as discussed in detail in Results and Discussion (Sec. [Sec sec3]).

## 3 Results and Discussion

### 3.1 Microcavity-Assisted Blackbody Infrared Radiative Dissociation via Doorway State

Our analysis of radiative dissociation in weakly coupled microcavities assumes a single diatomic molecule (or a dilute sample at a low enough pressure that collisions can be neglected) in a planar microcavity. The tunable electromagnetic environment afforded by the microcavity allows BIRD rates to be either enhanced or suppressed relative to the free space, depending on which transitions are affected by the modified photon DOS.

In Figure 2a, BIRD rates in the weak coupling regime are shown relative to the free space rate at *T* = 400 K. For small microcavity lengths *L*_C_ < 25 μm, a mild but consistent enhancement of BIRD rates is observed. At these lengths, the observed enhancement is associated with an overall increase in the microcavity electromagnetic DOS over a wide frequency range covering several important transitions. The ratios of microcavity BIRD rates to free space BIRD rates approach 1 as *L*_C_ increases beyond a few tens of micrometers. This is expected since the microcavity DOS converges to the free space limit when *L*_C_ → *∞*.

**Figure 2:**
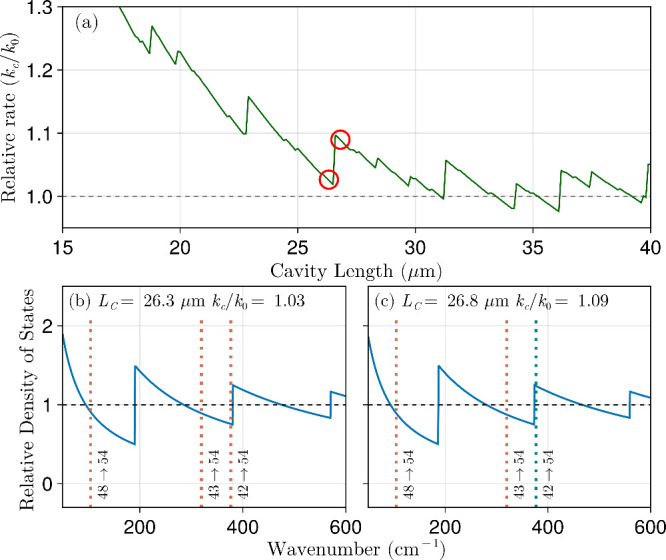
(a) Ratio of BIRD rate inside a nearly empty microcavity (weak coupling regime) to the free-space rate as a function of microcavity length for NaLi. Red circles highlight specific lengths further discussed in panels b and c. Microcavity photon DOS normalized to the free space DOS for *L*_C_ = 26.3 and 26.8 μm, in panels b and c, respectively. The dotted vertical lines indicate the location of the molecular transition frequencies corresponding to the most relevant overtone transitions, with color coding to distinguish between suppression (red) and enhancement (green).

On top of the main feature described above, we also observe an oscillatory pattern in the BIRD rates relative to free space shown in Figure 2a. This pattern arises from the interplay of discrete vibrational transitions enhanced or suppressed depending on *L*_C_. As we demonstrate below, this complexity is caused by overtones, which play an essential role in our analysis.

We conducted a sensitivity analysis of the dissociation rate to interpret the origin of the oscillatory behavior in Figure 2a and identify the most consequential radiative transitions involved in the BIRD of NaLi. This analysis measures how impactful an enhancement in the photon DOS at a specific transition frequency is on the dissociation process (see Supporting Information for more details). For NaLi dissociation, changes in the photon density of states corresponding to the frequency of overtone transitions *i* → 54, with *i* ∈ {42, 43, 48} are the most consequential for the BIRD rate. The identified overtones take the vibrational mode to the highest energy bound state, from which dissociation rapidly occurs. We emphasize that overtones are crucial because at high excitation levels fundamental transitions (*i* → *i* + 1) involve very small energies and, therefore, have associated small thermal photon populations and reduced oscillator strengths. See Supporting Information for a brief discussion of the results obtained without overtone transitions.

Equipped with knowledge of the most relevant transitions for the investigated BIRD of NaLi via the most energetic bound state, we can explain the microscopic origin of the intricate pattern in Figure 2a by considering how the microcavity DOS changes around the previously mentioned overtones. Figure 2b,c presents the ratio of microcavity DOS to the free space for selected values of *L*_C_ where suppression or enhancement of BIRD was verified in Figure 2a (red circles). Overtone frequencies are highlighted with dotted vertical lines color-coded by whether they are enhanced (green) or suppressed (red) in the corresponding microcavity. In Figure 2b (*L*_C_ = 26.3 μm), the most relevant NaLi overtones are suppressed, and this leads to the local minimum observed in *k*_c_/*k*_0_ in Figure 2. In contrast, when the microcavity length slightly increases to *L*_C_ = 26.8 μm (Figure 2c) the photon DOS is enhanced at the 42 → 54 transition frequency, and we observe a corresponding leap in the BIRD rate in Figure 2a. Introducing a finite line width to the considered radiative transitions would smooth out the oscillations observed in Figure 2a and dampen their amplitude. Consequently, the weakly coupled microcavity effect on BIRD would be significant only at lengths less than a few tens of micrometers.

Note that suppression effects are generally minor in Figure 2a because the dissociative process has many pathways. Therefore, blocking or slowing a particular transition may reduce the dissociation rate, but the effect is almost always negligible, as the molecular system explores alternative dissociation pathways. Our analysis of BIRD in weak coupling reveals two main results that we expect to hold generically for chemistry in microcavities: (i) overtone transitions are crucial and should not be ignored, and (ii) blocking a single reactive pathway is likely inconsequential. We conclude this section by noting that BIRD simulations with lossy mirrors (SI Sec. 8) show (i) broadening and suppression of the sharp features in Figure 2 and (ii) enhanced dissociation rates relative to Figure 2a at short *L*_C_ due to the presence of evanescent modes with significant amplitude near the imperfect metal interfaces.^7,61^ Overall, lossy mirrors suppress the oscillatory behavior of the relative BIRD rate with microcavity length while preserving the order-of-magnitude of the effects predicted for perfect mirrors. This confirms that, after averaging over the fast oscillations in Figure 2a, the results presented in this section are representative of experimentally accessible photon resonators.

### 3.2 Polariton-Assisted Blackbody Infrared Radiative Dissociation via Doorway State

To analyze polariton-assisted BIRD, we considered a microcavity containing, in addition to the reactive diatomic molecule, a host material strongly coupled to the resonator modes. Figure 1d illustrates this scenario, emphasizing the formation of polariton states, which effectively change the photon frequency–momentum dispersion relation (Figure 1e) and the electromagnetic DOS. Importantly, the reactive NaLi molecule remains weakly coupled to the confined electromagnetic field. We explore the implications for diatomic BIRD via the most energetic bound state in the following.

Figure 3 presents the ratio of polariton-assisted BIRD to the corresponding free space rates (*k*_p_/*k*_0_) as a function of the host matter frequency, ω_M_, and fixed microcavity length, *L*_C_, at 26.8 μm where we observed enhanced rates. In Figure 3a, we observe prominent peaks where the relative rates are notably enhanced. These peaks occur when the matter frequency (ω_M_) is greater than but sufficiently close to the specific overtone transition frequencies (ω_*i*→*j*_). The asymmetry in the relative dissociation rate profile around the observed maxima, shown in more detail in the inset of Figure 3a, emerges as a result of the formation of the polariton stopgap region. When ω_M_ is close to but smaller than ω_*i*→*j*_, the i→j transition falls within the stopgap region and is completely suppressed, as the density of states becomes zero at that frequency. Nonetheless, as discussed for the weak coupling regime, a strong suppression of reaction rates is not observed due to the many alternative pathways the reaction can access. Focusing on the 42 → 54 overtone (ω_42→54_ = 380 cm^–1^) which is the most consequential transition for the reaction, the effect of a variation in ω_M_ – ω_42→54_ at various collective coupling strengths (Ω_R_) on BIRD rates is examined in Figure 3b. Increasing Ω_R_ leads to a significant enhancement of the polariton-assisted BIRD rates, reaching a factor of 40 at the large Rabi frequency Ω_R_ = 200 cm^–1^ and small positive ω_M_ – ω_42→54_. As this frequency difference increases, the enhancement drops: at 3 cm^–1^, the enhancement is about 12-fold; at 5 cm^–1^ detuning, it is around 7-fold; and beyond 7 cm^–1^, the enhancement persists around 4-fold. This reduction in dissociation rate enhancement with increasing ω_M_ – ω_42→54_ follows from the behavior of the photon-weighted polariton DOS near the stopgap, as we discuss next. In Figure 4, the photon-weighted polariton density of states, *D*_P_(ω), is shown for selected values of Ω_R_ at *L*_C_ = 26.8 μm and ω_M_ = ω_42→54_ = 380 cm^–1^. The case where Ω_R_ = 0, representing the weak coupling limit, is included here for direct comparison with the empty microcavity density of states *D*_C_(ω). It can be seen from Figure 4 that the photon-weighted polariton DOS is only significantly different from the bare microcavity DOS in a frequency range around ω_M_ defined as {ω_M_ – α­(Ω_R_) < ω < ω_M_ + α­(Ω_R_)}, where α­(Ω_R_) is a positive frequency that depends on Ω_R_ and satisfies *D*_P_[ω_M_ ± α­(Ω_R_)] ≈ *D*_C_[ω_M_ ± α­(Ω_R_)]. Outside this range, *D*_P_(ω) retains features of the weak coupling case, such as oscillations in ω, which can be understood from eq 9. Figure 4 further illustrates how increasing Ω_R_ broadens the stopgap region and expands α­(Ω_R_), leading to a wider frequency range for which *D*_P_(ω) ≫ *D*_C_(ω). As a result, depending on the Rabi splitting, strong coupling can induce changes in the rates of multiple vibrational transitions.

**Figure 3:**
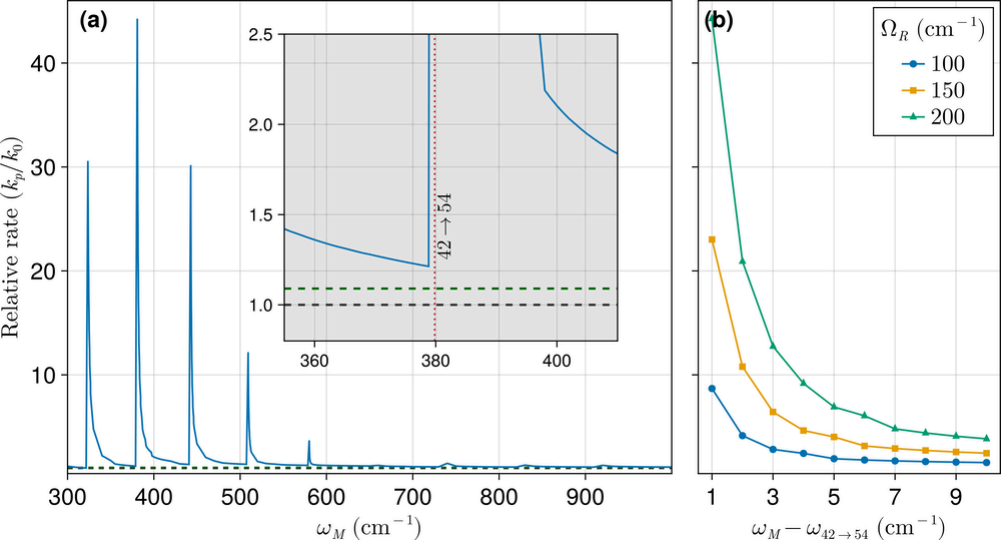
Ratio of polariton-assisted BIRD rates to free space BIRD rates. In the polaritonic case, the diatomic molecule is embedded in a strongly coupled microcavity with variable host material with frequency ω_M_. In panel a, the collective light–matter interaction strength, Ω_R_, is fixed at 200 cm^–1^. The horizontal dashed gray and green lines indicate where the dissociation rates equal *k*_0_ and *k*_c_, respectively. The minimum difference between ω_M_ and ω_*i*→*j*_ used here is 1 cm^–1^ to match panel b. The inset in panel a shows a zoomed-in view around the overtone 42 → 54. In panel b, relative BIRD rates are shown for different Rabi frequencies (Ω_R_), depicting the variation of the relative rate enhancement with the detuning between the host molecule and the diatomic transition energies.

**Figure 4:**
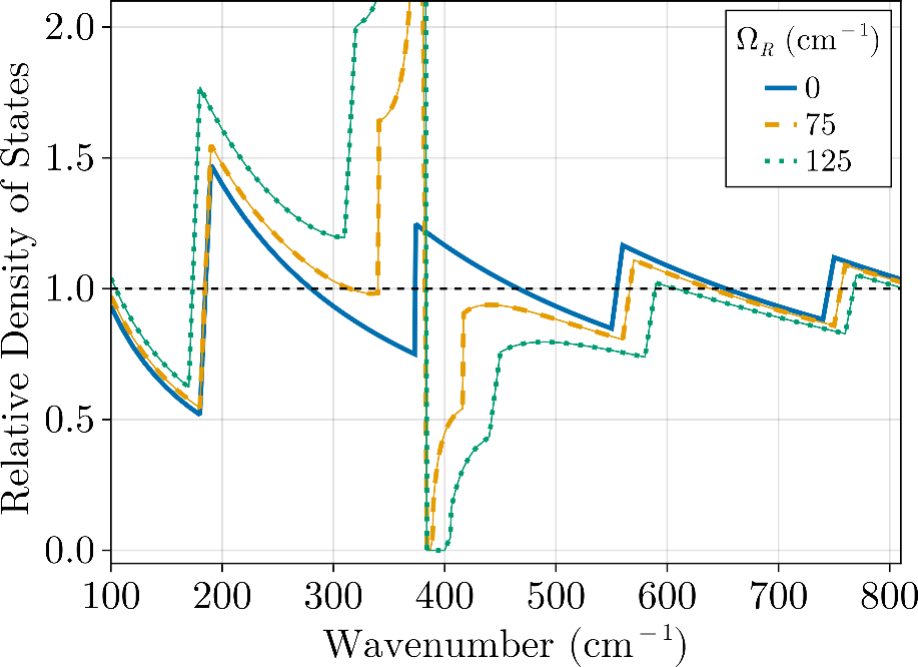
Photon-weighted polariton density of states *D*_P_(ω), normalized to the free space DOS, for a microcavity with length *L*_C_ = 26.8 μm strongly coupled to a material with a bright transition at frequency ω_M_ = 380 cm^–1^. The curves show the effect of different Rabi splitting values (Ω_R_) on the photon-weighted polariton DOS, which is relevant for radiative processes mediated by polaritonic systems. As Ω_R_ increases, the frequency range below ω_M_, where the photon-weighted polariton DOS significantly exceeds that of free space, widens. This trend explains the impact of the collective light–matter interaction strength on the observed polariton-assisted bond infrared dissociation (BIRD) enhancement shown in Figure 3.

The singular behavior of *D*_P_(ω) at ω_M_ is originated by the implicit assumption of our model that off-resonant photons with arbitrarily large energy mix with the strongly coupled matter system excitations and form polaritons. These interactions, albeit weak, lead to the formation of lower polariton (LP) modes with very small photon contents and frequencies arbitrarily close to those of ω_M_. This behavior is reflected in the expression for the photon-weighted polariton DOS in eq 12, where the photon content appears in the numerator and the polariton group velocity appears in the denominator. As the frequency ω approaches ω_M_, the group velocity vanishes faster than the photon content, resulting in an arbitrarily large photon DOS. This divergence in the DOS manifests itself as an accumulation of LP states near ω_M_, driven by the off-resonant interaction between matter and the microcavity radiation field. In other words, as the in-plane wave vector *k* increases, the LP transition frequencies approach ω_M_, yet they never become exactly equal in our model due to the presence of the stopgap. Consequently, within any arbitrarily small frequency interval ω_M_ – δ < ω < ω_M_, δ > 0, a large number of LP states exist, leading to a singularity in the photon-weighted polariton DOS at ω_M_.

The off-resonant light–matter interactions responsible for the singularity of *D*_P_(ω) at ω_M_ can be eliminated by imposing an effective frequency cutoff for polaritons formed from high-energy microcavity modes by setting a minimum nonvanishing value of δ = ω_M_ – ω_42→54_. For example, when δ = 10 cm^–1^, photon modes across 6 branches with ω_C_(*m*, *q*) = 945 cm^–1^ contribute to *D*_P_(ω), while when δ = 1 cm^–1^, *D*_P_(ω) contains contributions from 15 microcavity branches with ω_C_(*m*, *q*) = 2780 cm^–1^. Therefore, by choosing a nonvanishing threshold for δ, we also set a maximum energy (cutoff) for radiation states that contribute to the photon-weighted polariton DOS. Figure 3 shows that the removal of an increasing number of polaritons formed from highly off-resonant photons reduces the relative BIRD rate enhancement to an extent that depends on the Rabi frequency and the minimum δ. In experiments, imperfections in the strongly coupled material system and in the electromagnetic device (SI, section 8) smooth the photon-weighted DOS near the stopgap. Nonetheless, previously reported simulations show that the enhancement of *D*_P_(ω) persists around ω_M_ in the presence of an ultraviolet (UV) cutoff and material disorder.^70^ The introduction of a UV cutoff, cavity loss, and dephasing pathways for the matter polarization field are therefore expected to lead to reduced BIRD enhancements relative to those in Figure 3 depending in a nonuniversal way on Ω_R_ and parameters characterizing the quality of the strongly coupled subsystems (e.g., microcavity quality factor, matter homogeneous line width, etc.). Still, given the modest impact of leaky mirrors on the order of magnitude of the microcavity effect on the BIRD rates discussed in Sec. [Sec sec3.1] and SI Sec. 8, we expect the order of magnitude of polariton-assisted BIRD enhancement to be similarly robust to the introduction of microcavity losses characteristic of experimentally accessible devices.

Note that the singularity in the photon-weighted polariton DOS at ω_M_ enables a particular radiative transition to become much faster than the corresponding free space rate. However, it does not lead to arbitrarily fast reaction rates since the complex multistep reactive process ensures that other transitions quickly become rate-limiting (see the Supporting Information for a saturation analysis). Therefore, the reported BIRD rates obtained when ω_M_ approaches selected ω_*i*→*j*_ (Figure 3) provide a *finite* upper bound for polariton-enhanced BIRD rates in the lossless limit discussed here.

Before ending this section, we emphasize our observation of the same qualitative behavior for polariton-mediated BIRD in other diatomic species (see SI). Since these chemical species have significantly different energetics, we anticipate the main trends reported in this work also apply to other diatomic and polyatomic molecules, particularly in cases where dissociation occurs through a specific doorway state.

### 3.3 Dissociation via Radiative Bound-Continuum Transitions

In previous sections, we examined BIRD mediated by the most energetic bound-state. Gas-phase dissociation may also be promoted by direct decay from any bound-state *i* ≤ *i*_max_ into the dissociative continuum. This scenario is examined in this section, where we generalize our previous treatment to include direct (irreversible) dissociation pathways for each bound-state *i* with rate *k*_loss_^*i*^ as described in Methods (Sec. [Sec sec2]) and Supporting Information.

Figure 5a shows the relative dissociation rate, *k*_C_/*k*_0_, for a diatomic molecule inside a nearly empty microcavity as a function of its length. In contrast to the doorway mechanism discussed in Sec. [Sec sec3.1], which exhibits pronounced oscillations, the behavior here is noticeably simpler yet may be understood with the same tools previously employed to rationalize the observed trends in Figure 2. For example, from the normalized photon DOS at microcavity lengths *L* = 10.5 μm and *L* = 15.5 μm presented in Figure 5b,c corresponding to local minimum and maximum in Figure 5a, respectively, we observe that the ten most influential transitions, identified through sensitivity analysis, lie between 330–420 cm^–1^. At *L* = 10.5 μm, these transitions fall within a frequency range where the DOS is suppressed relative to free space, whereas at *L* = 15.5 μm, they match with a region of enhanced DOS. This variation accounts for the observed changes and local extrema in the microcavity dissociation rates in Figure 5a. Note when the diatomic dissociation occurs exclusively via the doorway state (as in Sec. [Sec sec3.1]), a few overtone transitions transferring population to the highest energy bound state control the reaction rate. However, when dissociation can proceed directly from each bound state, sensitivity analysis shows that numerous Δ*v* = +2 transitions (e.g., 12 → 14, 13 → 15, and 14 → 16) can have a significant impact on the reaction rate. This feature challenges the assignment of dominant reaction pathways, yet relaxes the conditions for enhancement or minor suppression of BIRD in a weakly coupled microcavity.

**Figure 5:**
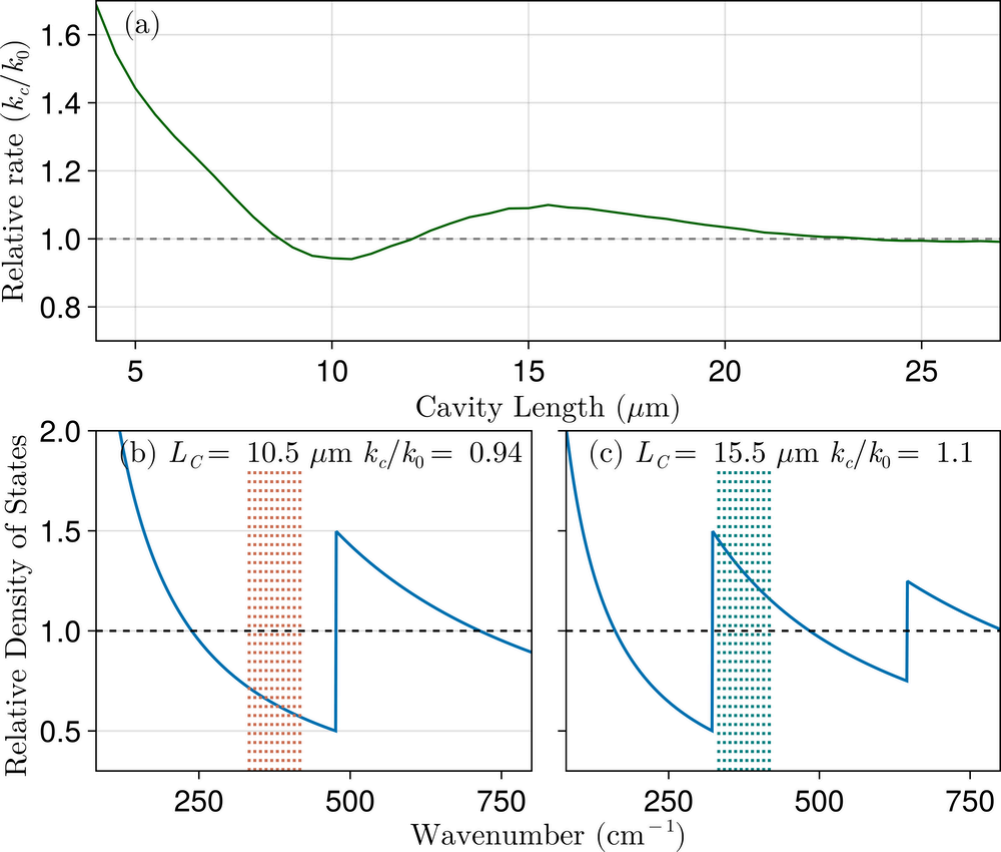
(a) Ratio of BIRD rate inside a nearly empty microcavity (weak coupling regime) to the free-space rate as a function of microcavity length for NaLi. Microcavity photon DOS normalized to the free space DOS for *L*_C_ = 10.5 and 15.5 μm, in panels b and c, respectively. The dotted vertical lines indicate the location of the molecular transition frequencies corresponding to the most relevant overtones, with color coding to distinguish between suppression (red) and enhancement (green).

Figure 6a presents the relative polariton-mediated BIRD rate, *k*_P_/*k*_0_, as a function of the host material frequency ω_M_ under strong coupling conditions with a collective light–matter interaction strength of Ω_R_ = 125 cm^–1^. Here, *L*_C_ is fixed at 5 μm, and reactant decay contributions from all vibrational states are included (unlike the results in Sec. [Sec sec3.2] which considered only decay from the highest bound state). In contrast to the isolated sharp peaks observed when the reaction proceeds via the doorway bound state (Figure 3), the introduction of multiple direct dissociative channels via bound-continuum transitions leads to a broader distribution of rate enhancements over a wide range of ω_M_. While resonant features persist in the reaction rate profile corresponding to particular overtone transitions (Figure 3a), the observed enhancements largely reflect cumulative contributions of decay channels proceeding directly from many intermediate vibrational bound states.

**Figure 6:**
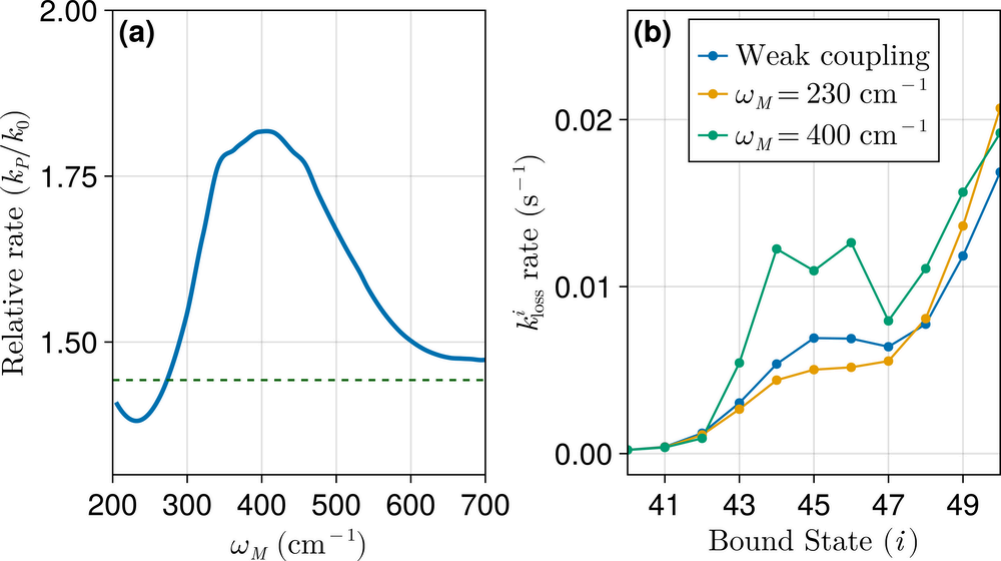
(a) Ratio of polariton-assisted BIRD rates to free-space BIRD rates for Ω_R_ = 125 cm^–1^. The horizontal dashed green line corresponds to the weakly coupled microcavity relative rate *k*_C_/*k*_0_. The solid line was computed using a LOESS moving average,^71–73^ which highlights the overall trend while smoothing out sharp resonance features. (b) Decay rates from bound vibrational states (*i* = 40–50) to the continuum under weak coupling (*L*_C_ = 5 μm) and under strong coupling for different ω_M_ values in the polaritonic environment.

To estimate the effect of broad vibrational line widths on the relative polariton-assisted BIRD rates, we applied locally estimated scatterplot smoothing (LOESS),^71–73^ a generalized moving average method. The resulting LOESS curve, shown as a solid line in Figure 6, reveals the underlying polaritonic enhancement of the BIRD rates while smoothing over sharp features that arise from approximating the continuum states of the Morse potential using a dense but discrete set of box states. This averaging is also supported by the presence of physical broadening mechanisms, including thermal rotational population and weak rovibrational interactions, which naturally contribute to infrared line widths.

The smoothed polariton-assisted BIRD rates reveal that at Ω_R_ = 125 cm^–1^, enhancement is possible when the host material frequency falls within the 300–500 cm^–1^ range. A mild suppression compared to the weak coupling case (*k*_P_/*k*_C_ < 1) is also possible when the host material frequency falls in a subset of the 200–300 cm^–1^ range. We attribute these features to the modification of bound-continuum transition rates (*k*_loss_^*i*^) as demonstrated in Figure 6b which shows these for bound vibrational levels near the dissociation threshold. When ω_M_ = 230 cm^–1^, the decay rates for certain intermediate bound states are slightly suppressed compared with weak coupling. In contrast, when ω_M_ = 400 cm^–1^, decay rates for states *i* = 43–47 are enhanced due to the modified photon weighted polariton DOS. Therefore, in Figure 6a, the minimum near ω_M_ = 230 cm^–1^ arises from suppressed decay channels due to a locally depleted polariton DOS, while the maximum near ω_M_ = 400 cm^–1^ results from faster vibrational decay via absorption into the continuum promoted by enhanced polariton DOS. When the reaction is allowed to proceed through this mechanism, which is sensible for gas phase systems, maximum enhancements are reduced compared to the BIRD mediated by the highest energy bound state (or any particular specific doorway state). However, demanding resonance conditions are also lifted, affording a more distributed enhancement profile that may offer a practical advantage for experimental realizations.

## 4 Conclusions

This work presents a detailed analysis of BIRD in infrared microcavities. Our analysis revealed mechanisms for controlling dissociation phenomena in photonic resonators, particularly by manipulating the electromagnetic density of states (DOS) in weak and strong light–matter coupling regimes. Overtones play a critical role in the dissociation process under both weak and strong coupling cases, as the corresponding transitions act as rate-limiting steps for the dissociative process occurring via the highest energy-bound state. The radiative dissociation rate changes induced by a microcavity are essentially tied to how the photonic density of states is modified around specific overtone transitions, driving the system into the dissociating (doorway) state.

We reported microcavity-assisted dissociation rate enhancements of *O*(1) in the weak coupling regime. In microcavities where *L*_C_ ≤ 25 μm, the photon DOS is greater than that in free space for most relevant diatomic transitions, leading to a mild increase in dissociation rates. As *L*_C_ increases beyond a few tens of micrometers, a complex pattern emerges from the interplay between enhanced and suppressed vibrational transitions, which can be rationalized by analyzing a few important overtones. Further increases in *L*_C_ cause the BIRD rates to approach the free-space limit.

In the strong coupling regime, where polaritons mediate BIRD, we reported *O*(10) enhancements when the host matter frequency is resonant with the rate-limiting overtones of the dissociative process. This enhancement is due to the emergence of a Rabi frequency dependent enhanced photon-weighted polariton DOS at a targeted frequency, which enables the increase of transition rates between levels with energy differences that approach the low-energy boundary of the polariton stopgap from below. The largest enhancements are obtained when photon modes highly off resonant with the host molecular system are assumed to form polaritons, yet as we showed, sizable polariton-assisted BIRD rate enhancements remain achievable when these highly off resonant interactions are cutoff to an extent that depends on Ω_R_ and parameters controlling losses and disorder. Ultimately, the BIRD enhancement factors here reported must be viewed as upper bounds for selected polariton-induced diatomic thermal radiative dissociation via the most energetic bound state.

The inclusion of bound-to-continuum transitions in our analysis alters the dissociation dynamics compared with the case where the reaction proceeds via a doorway state (the highest energy bound state). Dissociation via radiative bound-continuum transitions reveals a broader and smoother enhancement profile under microcavity length variation in the weak coupling regime. While the magnitude of the enhancement remains similar, the key difference lies in the involvement of a much greater number of vibrational transitions, relative to the doorway mechanism. In the polariton-assisted scenario, the relative BIRD rate enhancement is generally smaller but it no longer requires precisely matching a particular diatomic transition frequency to the host (strongly coupled) material. Instead, the multiplicity of direct dissociation pathways afforded by bound-to-continuum transitions makes polariton-enhanced BIRD inherently more robust, enabling broadband enhancement across a wide spectrum of host material frequencies ω_M_.

Our work focused on how optical microcavities can modify diatomic blackbody infrared radiative dissociation. While our analysis ignores collisional energy transfer and therefore may be viewed as providing upper bounds to the microcavity effect on diatomic BIRD rates, the main qualitative conclusions expressed above are expected to apply broadly to systems where thermal radiation acts as a relevant energy source. We anticipate that the framework developed here will aid in understanding and controlling thermal radiative-induced dissociation processes in more complex polyatomic systems.

## Supplementary Material





## Notes

Results for the HF and LiH molecules, temperature analysis, and additional computational details, including derivations, model parameters, and sensitivity analysis (PDF)
Transparent Peer Review report available (PDF)
