# Supplementary material for: Polaritonic Control of Blackbody Infrared Radiative Dissociation
Source: J Phys Chem Lett. 2025 Jul 18;16(30):7530–9. doi: 10.1021/acs.jpclett.5c01475 (PMC12319914; doi:10.1021/acs.jpclett.5c01475)
Supplement: Supplementary file 1 [file jz5c01475_si_001.pdf]

Supporting Information

Polaritonic control of blackbody infrared radiative dissociation

Enes Suyabatmaz,† Gustavo J. R. Aroeira,‡ and Raphael F. Ribeiro*,‡

†*Department of Physics, Emory University, Atlanta, GA, 30322*

‡*Department of Chemistry, Emory University, Atlanta, GA, 30322*

E-mail: raphael.ribeiro@emory.edu

Contents

List of Figures **4**

List of Tables **7**

1 Model parameters for Diatomic Molecules **8**

2 Rate Constant from Master Equation **13**

3 Decay Rates from a Bound Vibrational Level to Continuum States **15**

4 Free space thermal radiation **18**

5 Empty microcavity thermal radiation **19**

6 Microcavity thermal radiation and density of states under strong light-matter coupling **21**

7 HF and LiH molecules **28**

8 Dissociation Rates in Lossy Cavities **33**

8.1 Computational Method. 33

8.2 Results 36

8.3 Comment about polariton-assisted mechanism in lossy resonators 38

9 Sensitivity Analysis **39**

10 Temperature Dependence **42**

10.1 Temperature Dependence of a Model System 43

10.1.1 Low Temperature Free-space limit 46

10.1.2 Low Temperature Polaritonic limit 47

10.1.3 Temperature dependence of rate ratios 47

11 Rates without Overtones **51**

References **53**

List of Figures

S1 Morse potential and energy levels (shown as horizontal lines) used in this work for NaLi. Selected vibrational wave functions are also shown. They are shifted according to their corresponding energy for better visualization. 9

S2 Dipole functions for HF, LiH, and NaLi. For HF, we use a model function ($\mu(r) = \mu_0 r e^{-\zeta r^4}$) described in ref. 1. For LiH and NaLi, dipole moments were calculated using *ab initio* quantum chemistry, and interpolation was used to obtain smooth functions. Dashed vertical lines indicate the equilibrium radius for HF (0.926 Å), LiH (1.595 Å), and NaLi (2.895 Å). 12

S3 Decay rates from bound states to the continuum for different cavity lengths. The decay rate k_i increases significantly for higher initial bound states, especially in shorter cavities (5 μm). 17

S4 Ratio of BIRD rate inside a microcavity (weak coupling regime) to the free-space rate as a function of microcavity length for HF and LiH molecules. Red circles highlight specific lengths further discussed in Fig. S5. 28

S5 Microcavity photon DOS normalized to the free space DOS for selected lengths (L_C) where BIRD suppression and enhancement were observed for HF (left panels) and LiH (right panels) in Fig. S4. The dotted vertical lines indicate the location of the transition frequencies corresponding to the most relevant overtone transitions, with color coding to distinguish between suppression (red) and enhancement (green). Panel (a) shows that the small HF BIRD suppression observed at $L_C = 3.03$ μm is at least in part caused by the reduced relative DOS at the highlighted overtones. Panel (b) shows the mild enhancement of HF BIRD in a microcavity with $L_C = 3.21$ μm can be ascribed to the greater microcavity photon DOS at the frequencies of the highlighted overtones. Panels (c) and (d) show the analogous scenario for LiH, where BIRD is observed to be suppressed at $L_C = 9.4$ μm but enhanced at $L_C = 9.6$ μm, primarily due to the variation of the microcavity photon density of states at the selected $i \to 29$ overtone frequencies. 29
S6 Ratio of polariton-assisted BIRD rates to free space BIRD rates. In the polaritonic case, the diatomic molecule is embedded in a strongly coupled microcavity with variable host material with frequency ω_M. The top panels, (a) and (b), correspond to HF, and the bottom panels, (c) and (d), show results for LiH. In (a) and (c), the collective light-matter interaction strength, Ω_R, is fixed at 400 and 200 cm^{-1}, respectively. The horizontal dashed gray and green lines indicate where the dissociation rates equal k_0 and k_c, respectively. The insets in (a) and (c) show zoomed-in views around the overtones $17 \to 23$ for HF and $22 \to 29$ for LiH. In (b) and (d), relative BIRD rates are shown for different Rabi frequencies (Ω_R), depicting the dependency on the detuning between the host molecule and the diatomic transition energies. 31

S7 Photon-weighted polariton density of states $D_P(\omega)$, normalized to the free space DOS, for a microcavity with length $L_C = 9.6$ μm strongly coupled to a material with a bright transition at frequency $\omega_M = 1401$ cm^{-1}. The curves show the effect of different Rabi splitting values (Ω_R) on the photon-weighted polariton DOS, which is relevant for radiative processes mediated by polaritonic systems. As Ω_R increases, the frequency range below ω_M, where the photon-weighted polariton DOS significantly exceeds that of free space, widens. This trend explains the impact of the collective light-matter interaction strength on the observed polariton-assisted bond infrared dissociation (BIRD) enhancement shown in Fig. S6. 32

S8 Isotropically and spatially averaged LDOS $\langle D_C \rangle_{z_0}/D_0$ at $L = 25$ μm for perfect mirrors and three metal mirrors (Au, Al, Pt). Realistic metals suppress and broaden the modal features seen in the ideal case. 36

S9 Relative BIRD rate k_c/k_0 versus cavity length L at $T = 400$ K. Mirror materials include gold (Au), aluminum (Al), and an idealized perfect reflector. Lossy mirrors broaden and suppress the resonances, but maintain rate enhancements at shorter lengths. 37

S10 Sensitivity analysis for NaLi under enhancement ($\delta = 1.5$) and suppression ($\delta = 0.5$). The sensitivity was calculated using Eq. 71 following the procedure described in this section. Transitions not shown in the plot have negligible sensitivity. 41

S11 Temperature dependence of the relative BIRD rates for LiH in the **(a)** weak and **(b)** strong coupling regimes. In **(b)**, $\Omega_R = 200$ cm^{-1} and $L_C = 9.6$ μm . 44

S12 Minimal systems with two and three levels for BIRD rates are analytically examined. 45

S13 Comparison between BIRD rates obtained via numerical diagonalization and approximations derived in Eqs. 81 and 82. 48

S14 Polariton enhancement factors measured by the ration between rates with $\alpha \gg 1$ and $\alpha = 1$ computing using (a) approximate expression given in Eq. 91 and (b) numerical diagonalization of the transport matrix described in Eq. 73 . 50

S15 Ratio of BIRD rate, computed without overtones, inside a microcavity (weak coupling regime) to the free-space rate as a function of microcavity length for HF and LiH molecules. Dotted green and pink lines represent the relative density of states, $D_C(\omega)/D_0(\omega)$, at $\omega = 149.8$ cm^{-1} and $\omega = 61.3$ cm^{-1}, respectively. 52

List of Tables

S1 Morse parameters used for diatomic molecules. 10

S2 Computed dipole values for NaLi at the CCSDT level of theory[2] 11

S3 Drude parameters used for dielectric function calculations. 34

S4 Most important transitions according to their sensitivity analysis score for NaLi. 40

S5 Absolute BIRD rates for LiH in free space (k_0), weak coupling regime (k_c), and strong coupling regime (k_p). In all cases, $L_C = 9.6$ μm, $\Omega_R = 200$ cm^{-1}, and $\omega_M = 1402$ cm^{-1}. 43

S6 Computed free space BIRD rates (k_0) with and without overtone transitions at $T = 2000$ and 4000 K for LiH and HF, respectively. 51

1 Model parameters for Diatomic Molecules

We use a Morse potential to describe the potential energy of the examined diatomic molecules,

$$V(r) = D_e \left(1 - e^{-\alpha(r-r_e)}\right)^2 , \tag{1}$$

where

$$\alpha = \sqrt{\frac{k_e}{2D_e}} , \tag{2}$$

$$k_e = m_r (2\pi c \nu_e)^2 , \tag{3}$$

with c being the speed of light in the vacuum, m_r the reduced mass and ν_e the harmonic frequency of the system. The energy levels are calculated as

$$E(n)/hc = \nu_e \left(n + \frac{1}{2}\right) - \nu_e \chi_e \left(n + \frac{1}{2}\right)^2 , \tag{4}$$

where n is the vibrational quantum number, h is Planck's constant and χ_e is the anharmonicity constant. The number of bound states is given by

$$n_{\max} = \left\lfloor \frac{\sqrt{2m_r D_e}}{\hbar\alpha} - \frac{1}{2} \right\rfloor . \tag{5}$$

For NaLi molecule, the dipole function and Morse parameters were extracted from previous CCSDT/cc-pCVQZ calculations available in the literature.[2] For the HF molecule, we obtained all Morse parameters from previous work on laser-induced dissociation.[1,3–5] For LiH molecule, harmonic and anharmonic frequencies were obtained from experimental data,[6] and the dissociation energy was extrapolated from these parameters using the following

relationship valid for a Morse potential:

$$D_e = \frac{hc\nu_e^2}{4(\nu_e \chi_e)} \, . \tag{6}$$

The dissociation energy (D_e) obtained using this method (2.641 eV) deviates slightly from reported experimental[6–8] and other *ab initio* computations[9–11] (approximately 2.515 eV). This discrepancy arises because the electronic ground-state LiH potential energy curve deviates from the Morse potential assumed for simplicity in Eq. 6. However, we emphasize that the parameters used for NaLi, HF, and LiH are representative of these species, and a more precise description would not change the conclusions of this work in any significant way. All used Morse potential parameters are collected in Table S1.

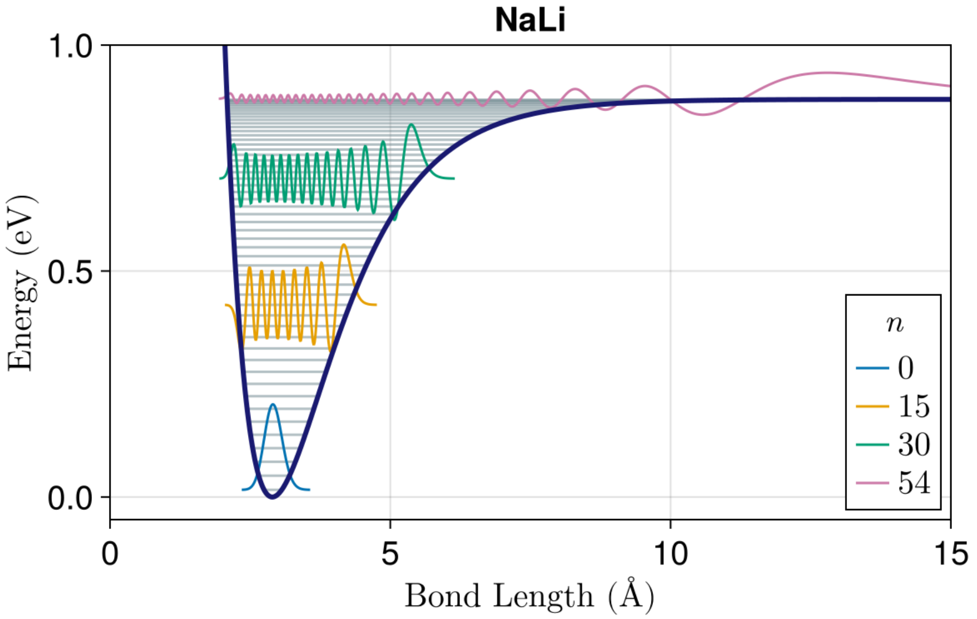

Figure S1: Morse potential and energy levels (shown as horizontal lines) used in this work for NaLi. Selected vibrational wave functions are also shown. They are shifted according to their corresponding energy for better visualization.

Morse potentials corresponding to the given parameters are shown in Fig. S1 along with selected wave functions. The wave functions are computed as described by Dahl and Springborg.[12] In Fig. S1, their extension is truncated when their amplitudes squared are

Table S1: Morse parameters used for diatomic molecules.

**Constant**	**HF**	**LiH**	**NaLi**
D_e (eV)	6.123	2.641	0.882
r_e (Å)	0.926	1.595	2.895
ν_e (cm^{-1})	4138	1405	257.4
$\nu_e \chi_e$ (cm^{-1})	86.70	23.162	2.33
m_r (a.u)	0.950	0.874	5.331

below 1×10^{-8}, giving us an idea of their spatial extent. It can be seen, for example, that in both cases, the final bound state ($n = 23, 29$ and 54 for HF, LiH and NaLi, respectively) spans a bigger domain than lower energy states. This has consequences when choosing an integration grid as we discuss next.

Electrical dipole transition matrix elements μ_{ij} can be computed from the vibrational wave functions as

$$\mu_{ij} = \int_{-\infty}^{+\infty} \psi_i^*(r)\mu(r)\psi_j(r)dr\,, \tag{7}$$

where $\psi_k(r)$ represents the k-th vibrational state and $\mu(r)$ describes the electronic dipole moment change dependence on r.

In the case of NaLi, the dipole function was constructed using a B-spline interpolation available in the DataInterpolations.jl package. The data used for the interpolation were obtained from previous CCSDT/cc-pCVQZ calculations.[2] For completeness, these values are shown in Table S2.

Fig. S2 shows the dipole function for NaLi. Calculating dipoles for small bond lengths becomes increasingly difficult due to convergence issues. This region of configuration space is irrelevant for the computation of transition dipole matrix elements since the vibrational wave functions decay exponentially at small r (see Fig. S1). Hence, we enforce $\mu \to 0$ in the interpolation as $r \to 0$. This has no impact on the computed transition dipole matrix

elements. Eq. 7 is evaluated numerically with integration domain spanning $r_{\text{min}} = 0.0$ Å to $r_{\text{max}} = 50$ Å with a grid spacing of 0.01 Å.

Table S2: Computed dipole values for NaLi at the CCSDT level of theory[2]

Bond length (Å)	Dipole moment ($e \cdot$ Å)
1.8	0.13137057268257263
2.0	0.10659545675669918
2.2	0.09764310394314826
2.5	0.0993086579549717
2.9	0.11138392454069153
3.5	0.130329601425183
3.8	0.13282793244291813
4.0	0.12991321292222713
4.2	0.12137724861163207
4.5	0.10805281651704468
5.0	0.07390895927466447
5.5	0.04330440430740904
6.0	0.023109561914050017
7.0	0.006037633292859915
8.0	0.0018737482633013527
10.0	0.00062458275443337843
12.0	0.00062458275443337843
15.0	0.00062458275443337843

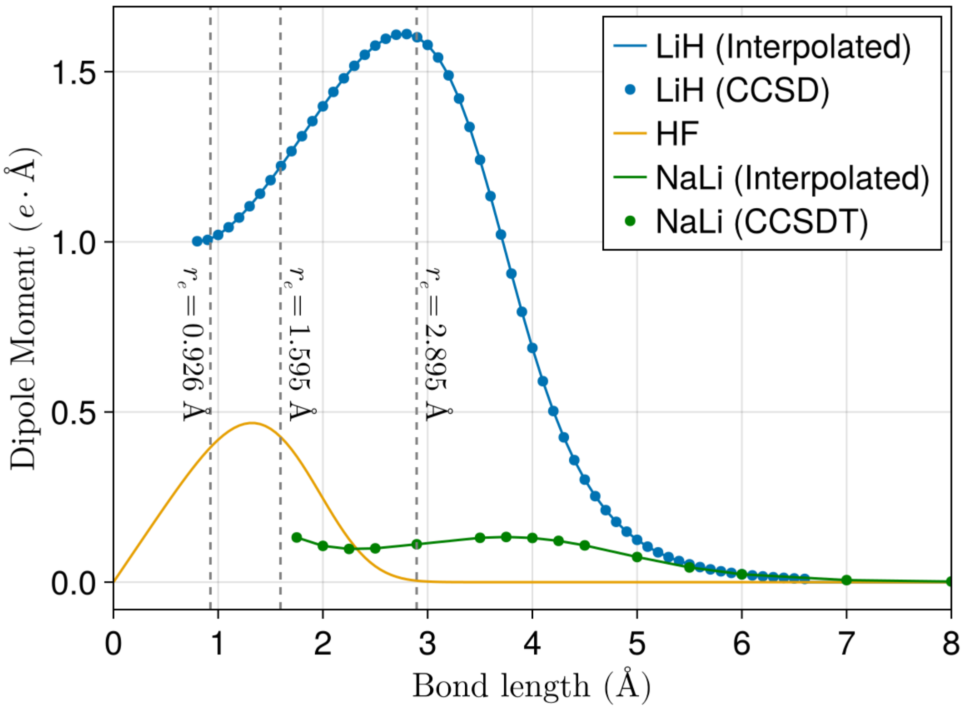

Figure S2: Dipole functions for HF, LiH, and NaLi. For HF, we use a model function ($\mu(r) = \mu_0 r e^{-\zeta r^4}$) described in ref. 1. For LiH and NaLi, dipole moments were calculated using *ab initio* quantum chemistry, and interpolation was used to obtain smooth functions. Dashed vertical lines indicate the equilibrium radius for HF (0.926 Å), LiH (1.595 Å), and NaLi (2.895 Å).

2 Rate Constant from Master Equation

The dynamics of the reactive molecule is modeled with a Markov Master equation routinely employed in studies of blackbody infrared radiative molecules in free space.[13] In this formalism, the time evolution of the vibrational population vector $[\mathbf{N}] = [N_1, N_2, ..., N_n]$ is described by the equation

$$\frac{d[\mathbf{N}]}{dt} = -\mathbf{J} \cdot [\mathbf{N}], \tag{8}$$

where N_ν is the population of state ν, $n = n_{\max}$ (Eq. 5), and $\mathbf{J}$ is the transition matrix

$$\mathbf{J} = \begin{pmatrix} (k_{12} + \cdots + k_{1n}) & -k_{21} & \cdots & -k_{n1} \\ -k_{12} & (k_{21} + \cdots + k_{2n}) & \cdots & -k_{n2} \\ \vdots & \vdots & \ddots & \vdots \\ -k_{1n} & -k_{2n} & \cdots & k_{\text{loss}} \end{pmatrix}. \tag{9}$$

Each matrix element J_{ij} with $i \neq j$ corresponds to the transition rate k_{ij} from state i to state j. Diagonal elements J_{ii} represent the total outgoing rate from state i. This includes all transitions from state i to every other state j. The diagonal elements are always positive. The last row of $\mathbf{J}$ includes k_{loss}, which represents the irreversible loss of population due to the dissociation in the highest energy bound level.

The eigenvalues of the transition matrix J are guaranteed to be positive,[14] and the smallest eigenvalue λ_1 of $\mathbf{J}$ is the rate constant of the unimolecular reaction. This is because, in the long-time limit, the system relaxes to the slowest decaying mode, i.e., the smallest eigenvalue of the transition matrix.

To show that the smallest eigenvalue gives the reaction rate constant, we note that the solution to Eq. 8 can be expressed as:[15]

$$[\mathbf{N}(t)] = \sum_{i=1}^{n} c_i \, e^{-\lambda_i t} [\mathbf{v}_i], \tag{10}$$

where λ_i are the eigenvalues of $\mathbf{J}$, and $[\mathbf{v}_i]$ are the corresponding eigenvectors. Let the eigenvalues be ordered as $\lambda_1 < \lambda_2 < \cdots < \lambda_n$. As $t \to \infty$, the term with the smallest eigenvalue λ_1 dominates the time evolution and all of the remaining $e^{-\lambda_2 t}, \ldots, e^{-\lambda_n t}$ approach zero rapidly because their corresponding λ_i values are larger than λ_1. The term $e^{-\lambda_1 t}$ gives the slowest decay, since λ_1 is the smallest. This means that the slowest decaying term effectively dominates the sum after a sufficiently long induction time $t \gg 1/\lambda_2$ leading to

$$[\mathbf{N}(t)] \sim c_1 e^{-\lambda_1 t}[\mathbf{v}_1], \ t \gg 1/\lambda_2 \tag{11}$$

where c_1 is a constant determined by the initial conditions. This shows that the population at any energy level decays at the same rate λ_1 and thus λ_1 is the unimolecular reaction rate.[16]

3 Decay Rates from a Bound Vibrational Level to Continuum States

To calculate the decay rate of a diatomic molecule from a bound vibrational level to continuum states above the dissociation threshold, we use Fermi's Golden Rule to describe the transition from a discrete bound state to a continuum of unbound, or scattering, states:

$$k^i_{\text{loss}} = \frac{2\pi}{\hbar} \sum_{f>\nu_{\max}} \left| \langle \psi_f | \hat{H}' | \psi_i \rangle \right|^2 \rho(E_f) \tag{12}$$

where $|\psi_i\rangle$ is the initial bound vibrational eigenstate, $|\psi_f\rangle$ is a final continuum state with energy E_f, $\hat{H}'$ represents the interaction Hamiltonian (typically the dipole operator $\hat{\mu}$ in IR-driven transitions), and $\rho(E_f)$ is the density of final states per unit energy. The summation over f effectively becomes an integral when the continuum is dense, and $\rho(E_f)$ accounts for the normalization of the scattering states.

We have an analytical form of the bound state wavefunction ψ_i, which for vibrational states can be obtained by solving the Schrödinger equation for a Morse potential. For the continuum state wavefunction ψ_f, scattering states that describe the free motion of the atoms after dissociation are often used. We calculate scattering states by numerical methods, such as the Numerov method,[17] and use them to compute transition dipole matrix elements and decay rates to the continuum. In practice, we approximate the continuum states of the Morse potential using a dense but discrete set of box states, allowing us to converge the k^i_{loss} rates numerically.

The BIRD kinetics via radiative bound-continuum transitions is modeled using a Master Equation framework similarly, where the time evolution of vibrational state populations is governed by both radiative transitions between bound states and irreversible loss to the dissociation continuum. In the presence of continuum states, each bound vibrational level can decay not only to other bound levels via stimulated absorption and emission, but also to a continuum of unbound states. This provides an additional decay pathway for each

vibrational state, characterized by the continuum loss rate k_i^{loss} derived from Eq. 12. The transition matrix $\mathbf{J}$ now includes bound-continuum decay rates on the diagonal elements:

$$\mathbf{J} = \begin{pmatrix} (k_{12} + \cdots + k_{1n} + k_1^{\mathrm{loss}}) & -k_{21} & \cdots & -k_{n1} \\ -k_{12} & (k_{21} + \cdots + k_{2n} + k_2^{\mathrm{loss}}) & \cdots & -k_{n2} \\ \vdots & \vdots & \ddots & \vdots \\ -k_{1n} & -k_{2n} & \cdots & (k_{n1} + \cdots + k_{n.n-1} + k_n^{\mathrm{loss}}) \end{pmatrix}. \tag{13}$$

The presence of k_i^{loss} on the diagonal ensures that population is irreversibly removed from each level due to dissociation. The matrix $\mathbf{J}$ remains positive-definite, and its smallest eigenvalue λ_1 continues to govern the long-time dynamics of the system. Specifically, the solution to Eq.8 in the asymptotic limit is:

$$[\mathbf{N}(t)] \sim c_1 e^{-\lambda_1 t} [\mathbf{v}_1], \tag{14}$$

indicating that the unimolecular dissociation rate constant is identified with the smallest eigenvalue λ_1 of the full transition matrix, now including continuum losses.

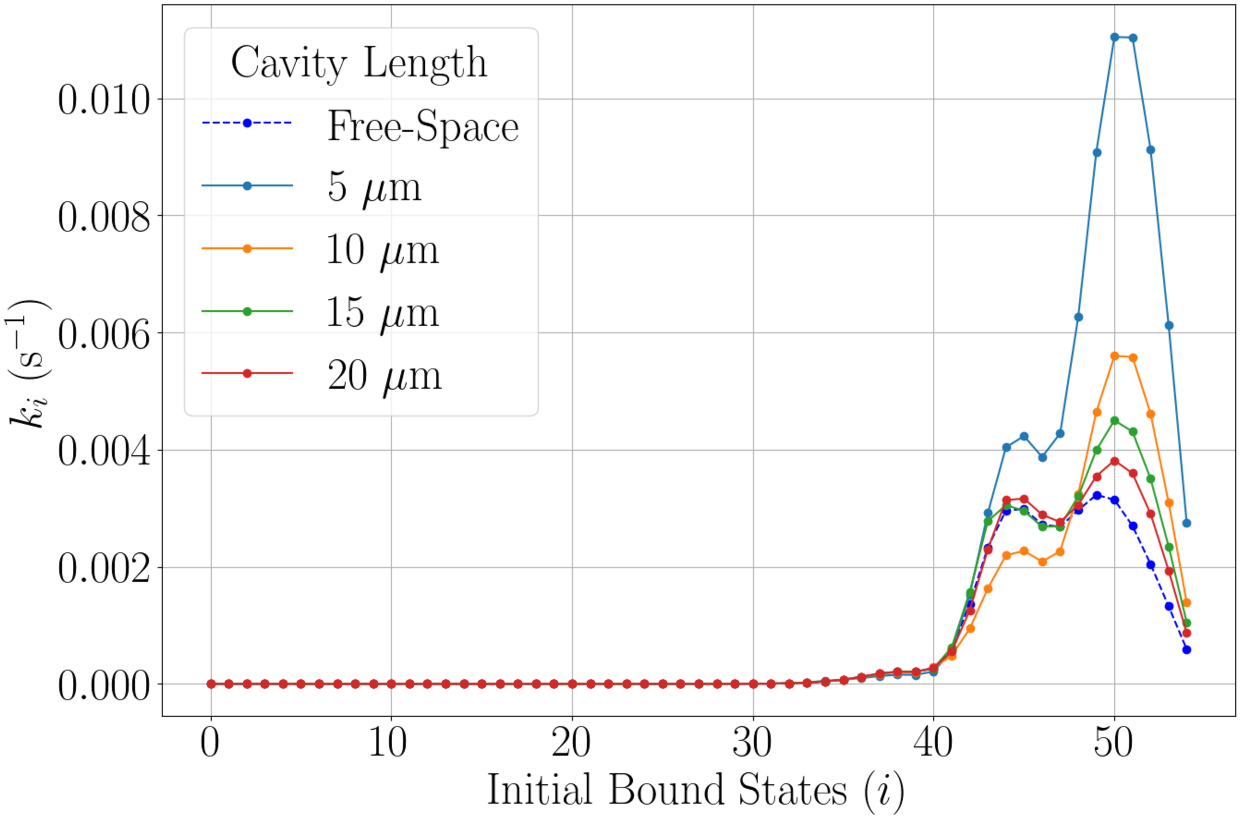

Figure S3: Decay rates from bound states to the continuum for different cavity lengths. The decay rate k_i increases significantly for higher initial bound states, especially in shorter cavities (5 μm).

4 Free space thermal radiation

The Hamiltonian for the free photon field, which describes the energy of a system of non-interacting photons, is given by:

$$H_0 = \sum_{\boldsymbol{k},\lambda} \hbar\omega_{\boldsymbol{k}} a^\dagger_{\boldsymbol{k},\lambda} a_{\boldsymbol{k},\lambda}, \tag{15}$$

where $\boldsymbol{k}$ is the wave vector, $\lambda = 1,2$ is the polarization, $\omega_{\boldsymbol{k}}$ is the angular frequency of the mode with wave vector $\boldsymbol{k}$, and $a^\dagger_{\boldsymbol{k},\lambda}$ and $a_{\boldsymbol{k},\lambda}$ are the creation and annihilation operators for the photon in that mode.

The free electromagnetic field partition function $Z(\beta) = \mathrm{tr}(e^{-H/k_\mathrm{B}T})$ allows us to compute all thermodynamic quantities of the bare field. The total radiation thermal energy at inverse temperature $\beta = 1/k_\mathrm{B}T$ is given by

$$E(T) = -\frac{\partial \log Z(\beta)}{\partial \beta} = \sum_{\lambda} \frac{\hbar\omega_k}{e^{\hbar\omega_k/k_\mathrm{B}T} - 1}, \tag{16}$$

and the mean photon number for a mode with frequency ω is given by

$$n(\omega, T) = \frac{1}{e^{\frac{\hbar\omega_k}{k_B T}} - 1}. \tag{17}$$

The density of photon states $D_0(\omega)$ corresponds to the number of available electromagnetic modes per unit frequency per unit volume at a frequency ω. For simplicity, we consider free space to be a three-dimensional periodic box with lengths $L_x = L_y = L_z = L$. The free space DOS is given by:[18]

$$D_0(\omega) = \frac{1}{V} \sum_{\boldsymbol{k},\lambda} \delta(\omega - ck) \tag{18}$$

where $V = L^3$. Using the limit where $L \to \infty$ and

$$\frac{1}{V} \sum_{\boldsymbol{k}} \to \frac{1}{(2\pi)^3} \int d^3k, \tag{19}$$

we obtain the free space photon DOS using standard manipulations

$$D_0(\omega) = 2\frac{4\pi}{(2\pi)^3}\cdot\frac{\omega^2}{c^3} = \frac{\omega^2}{\pi^2 c^3}. \tag{20}$$

The energy density $\rho_0(\omega,T)$ of free-field blackbody radiation can be obtained by multiplying the density of states with the Bose-Einstein distribution function and the energy of each photon:

$$\rho_0(\omega,T) = D_0(\omega)n(\omega,T) = \frac{\omega^2}{\pi^2 c^3}\cdot\frac{\hbar\omega}{e^{\frac{\hbar\omega}{k_B T}}-1}. \tag{21}$$

5 Empty microcavity thermal radiation

Consider an optical Fabry-Perot microcavity composed of two parallel perfect mirrors with area S separated by a distance L. The microcavity mode frequencies are given by

$$\omega_{\mathrm{C}}(m,q) = c\sqrt{q_x^2 + q_y^2 + \left(\frac{m\pi}{L}\right)^2}, \tag{22}$$

where $\mathbf{q} = (q_x, q_y)$ is the in-plane wave vector (momentum) corresponding to the in-plane momentum of the cavity modes, and m is zero (transverse magnetic polarization only) or a positive integer (for transverse electric and tranverse magnetic modes) representing the quantization of the wavevector in the z-direction (perpendicular to the mirror planes).[19,20] It follows the empty microcavity DOS is given by

$$D_{\mathrm{C}}(\omega) = \frac{1}{V}\sum_{m,\mathbf{q},\lambda}\delta[\omega - \omega_{\mathrm{C}}(m,q)], \tag{23}$$

where $V = LS$ is the resonator volume, λ denotes the polarization state, and m and $\mathbf{q}$ are as discussed above.

Using standard manipulations, we can write the total density of microcavity photon states

as[18]

$$D_C(\omega) = \frac{\omega}{\pi c^2 L} \left\lfloor \frac{\omega L}{\pi c} \right\rfloor + \frac{\omega}{2\pi c^2 L}, \tag{24}$$

where $\lfloor x \rfloor$ equals the greatest integer less than or equal to x. It follows the microcavity blackbody radiation energy density $\rho_C(\omega, T)$ is

$$\rho_C(\omega, T) = D_C(\omega) n(\omega, T) = \frac{\left\{ \left\lfloor \frac{\omega L}{\pi c} \right\rfloor + \frac{1}{2} \right\} \omega}{\pi c^2 L} \frac{\hbar \omega}{e^{\frac{\hbar \omega}{k_B T}} - 1}. \tag{25}$$

6 Microcavity thermal radiation and density of states under strong light-matter coupling

In the strong coupling regime, the Hamiltonian of the system is the sum of the Hamiltonians of the matter, field, and the matter-field interaction:

$$H_{\text{tot}} = H_{\text{L}} + H_{\text{SM}} + H_{\text{INT}}, \tag{26}$$

where each term is defined as follows: The free photon field Hamiltonian is

$$H_L = \sum_{\mathbf{q},m,\lambda} \hbar\omega_C(m,q)\left(a^{\dagger}_{m\mathbf{q}\lambda}a_{m\mathbf{q}\lambda} + \frac{1}{2}\right) \tag{27}$$

with in-plane wavevector $\mathbf{q} = (q_x, q_y)$, the mode number m, and the polarization index λ defined in Eqs. 22, 23. The matter Hamiltonian describes a homogeneous dispersionless isotropic material ensemble with significant infrared oscillator strength at a suitable frequency ω_M with effective Hamiltonian:[21]

$$H_M = \int_{D_M} \frac{d^3r}{v}\left[\frac{\boldsymbol{\pi}^2(\mathbf{r})}{2m^*} + \frac{m^*\omega_M^2\boldsymbol{\phi}^2(\mathbf{r})}{2}\right], \tag{28}$$

where D_M is the region of space occupied by the material, v is the volume occupied by a single local oscillator ("unit-cell volume"), m^* is the effective mass, $\mathbf{r} = (x, y, z) \in D_M$ and $\boldsymbol{\pi}(\mathbf{r})$ and $\boldsymbol{\phi}(\mathbf{r})$ represent the conjugate momentum and the matter displacement field respectively. The latter can be rewritten as:

$$\boldsymbol{\phi}(\mathbf{r}) = \sqrt{\frac{\hbar}{2m^*N\omega_M}} \sum_{\mathbf{q},m,\lambda}\left[b_{m\mathbf{q}\lambda}\mathbf{u}_{m\mathbf{q}\lambda}(\mathbf{r})e^{i\mathbf{q}\cdot\mathbf{r}_\parallel} + b^{\dagger}_{m\mathbf{q}\lambda}\mathbf{u}^*_{m\mathbf{q}\lambda}(\mathbf{r})e^{-i\mathbf{q}\cdot\mathbf{r}_\parallel}\right], \tag{29}$$

where $b_{m\mathbf{q}\lambda}$ and $b^{\dagger}_{m\mathbf{q}\lambda}$ are the annihilation and creation operators for the collective matter excitation labeled by $(m\mathbf{q}\lambda)$, $N = S/v$ denotes the number of molecular modes per unit

length, $\mathbf{u}_{m\mathbf{q}\lambda}(\mathbf{r})$ is the mode spatial profile vector and $\mathbf{r}_{\parallel}=(x,y)$. Each local oscillator vibration is triply degenerate. The excitations can be treated as bosonic following the standard dilute excitation limit. In this case, the Hamiltonian can be written in a simple way using second-quantization

$$H_M=\sum_{\mathbf{q},m,\lambda}\hbar\omega_M b^{\dagger}_{m\mathbf{q}\lambda}b_{m\mathbf{q}\lambda}. \tag{30}$$

We work in the dipole gauge, where the light-matter interaction is given by

$$H_{\text{int}}=\frac{1}{2\epsilon_0}\int_{D_M}\left[-2\mathbf{D}(\mathbf{r})\cdot\mathbf{P}(\mathbf{r})+\mathbf{P}^2(\mathbf{r})\right]d^3\mathbf{r}, \tag{31}$$

where $\mathbf{P}(\mathbf{r})$ is the matter polarization density given by:

$$\mathbf{P}(\mathbf{r})=\frac{eZ^*\boldsymbol{\phi}(\mathbf{r})}{v}, \tag{32}$$

where eZ^* is the effective charge, and $\mathbf{D}(\mathbf{r})$ is the electrical displacement field[22,23]

$$\mathbf{D}(\mathbf{r})=\sum_{\mathbf{q},m,\lambda}\sqrt{\frac{\epsilon_0\hbar\omega_C(m,q)}{2V}}\left(\mathbf{u}_{m\mathbf{q}\lambda}(\mathbf{r})a_{m\mathbf{q}\lambda}e^{i\mathbf{q}\cdot\mathbf{r}_{\parallel}}-\mathbf{u}^*_{m\mathbf{q}\lambda}(\mathbf{r})a^{\dagger}_{-m\mathbf{q}\lambda}e^{-i\mathbf{q}\cdot\mathbf{r}_{\parallel}}\right), \tag{33}$$

and $V=SL_C$ is the microcavity volume. Inserting the polarization operator expression in second-quantization representation into the light-matter interaction and separately writing linear and quadratic terms of the interaction Hamiltonian in the matter polarization field[24] we obtain

$$H_{\text{int},1}=i\sum_{\mathbf{q},m,\lambda}\frac{\hbar\Omega_R}{2}\sqrt{\frac{\omega_C(m,q)}{\omega_M}}\left(a^{\dagger}_{m\mathbf{q}\lambda}-a_{-m\mathbf{q}\lambda}\right)\left(b_{m\mathbf{q}\lambda}+b^{\dagger}_{-m\mathbf{q}\lambda}\right), \tag{34}$$

$$H_{\text{int},2}=\sum_{\mathbf{q},m,\lambda}\hbar\omega_M b^{\dagger}_{m\mathbf{q}\lambda}b_{m\mathbf{q}\lambda}+\frac{\hbar\Omega_R^2}{4\omega_M}\left(b^{\dagger}_{m\mathbf{q}\lambda}+b_{m-\mathbf{q}\lambda}\right)\left(b^{\dagger}_{m-\mathbf{q}\lambda}+b_{m\mathbf{q}\lambda}\right) \tag{35}$$

where $\Omega_R=\sqrt{\frac{(eZ^*)^2}{\epsilon_0 m^* v}}$ is the coupling constant, describing the strength of the interaction between the light field and the molecular excitations.

To find the normal modes of the light-matter system, we first define a new set of operators $p_{m\mathbf{q}\lambda}$ such that:

$$[p_{m\mathbf{q}\lambda}, H_M + H_{\text{int},2}] = \hbar\Omega_M p_{m\mathbf{q}\lambda}, \quad \Omega_M^2 = \omega_M^2 + \Omega_R^2 \tag{36}$$

where Ω_M represents the renormalized material excitation due to light-matter coupling inside the microcavity. The new polarization operators $p_{m\mathbf{q}\lambda}$ are written in terms of the original matter operators $b_{m\mathbf{q}\lambda}$ and their conjugates:

$$p_{m\mathbf{q}\lambda} = \frac{\Omega_M + \omega_M}{2\sqrt{\Omega_M\omega_M}} b_{m\mathbf{q}\lambda} + \frac{\Omega_M - \omega_M}{2\sqrt{\Omega_M\omega_M}} b^\dagger_{m-\mathbf{q}\lambda}. \tag{37}$$

This transformation effectively diagonalizes the quadratic part of the Hamiltonian, allowing us to describe the system in terms of new quasi-particles that reflect the modified nature of the molecular excitations under strong coupling. The total Hamiltonian, now expressed in terms of the new operators, takes the form:

$$\begin{aligned} H_{\text{total}} = &\sum_{\mathbf{q},m,\lambda} \hbar\Omega_M p^\dagger_{m\mathbf{q}\lambda} p_{m\mathbf{q}\lambda} + \sum_{\mathbf{q},m,\lambda} \hbar\omega_C(m,q)\left(a^\dagger_{m\mathbf{q}\lambda} a_{m\mathbf{q}\lambda} + \frac{1}{2}\right) \\ &+ i\sum_{\mathbf{q},m,\lambda} \hbar\frac{\Omega_R}{2}\sqrt{\frac{\omega_C(m,q)}{\Omega_M}}\left(a^\dagger_{m\mathbf{q}\lambda} - a_{-m\mathbf{q}\lambda}\right)\left(p_{m\mathbf{q}\lambda} + p^\dagger_{-m\mathbf{q}\lambda}\right), \end{aligned} \tag{38}$$

To obtain the polaritonic (hybrid) normal-modes after performing this initial matter Bogoliubov transformations, we note the total light-matter Hamiltonian can be written as:

$$H_{\text{total}} = \sum_{\mathbf{q},m,\lambda} \hbar\omega(m,q)\Pi^\dagger_{m\mathbf{q}\lambda}\Pi_{\mathbf{q}m\lambda}, \tag{39}$$

where $\Pi^\dagger_{\mathbf{q}m\lambda}$ and $\Pi_{m\mathbf{q}\lambda}$ are the creation and annihilation operators of polariton modes. In what follows, we simplify the notation by replacing $m\mathbf{q}\lambda$ in polariton operators by k. Polariton annihilation operators are given by

$$\Pi_k = x_k a_k + y_k a^\dagger_{-k} + z_k p_k + t_k p^\dagger_{-k}. \tag{40}$$

where x_k and y_k are the Hopfield coefficients representing the photonic contributions, and z_k and t_k are the coefficients representing the matter contributions. These coefficients are obtained by enforcing the bosonic commutation relations for the polariton operators and the condition $[\Pi_k, H_{\text{tot}}] = \hbar\omega(k)\Pi_k$ where $\omega(k)$ is a normal-mode (polariton) frequency. This leads to the set of linear equations.

$$|x_k|^2 - |y_k|^2 + |z_k|^2 - |t_k|^2 = 1, \tag{41}$$

$$[\omega(k) - \omega_C(m,q)]x_k + G\sqrt{\frac{\omega_C(m,\mathbf{q})}{\Omega_M}}(z_k + t_k) = 0, \tag{42}$$

$$[\omega(k) + \omega_C(m,q)]y_k + G\sqrt{\frac{\omega_C(m,q)}{\Omega_M}}(z_k + t_k) = 0, \tag{43}$$

$$[\omega(k) - \Omega_M]z_k - G\sqrt{\frac{\omega_C(m,q)}{\Omega_M}}(x_k - y_k) = 0, \tag{44}$$

$$[\omega(k) + \Omega_M]t_k + G\sqrt{\frac{\omega_C(m,q)}{\Omega_M}}(x_k - y_k) = 0, \tag{45}$$

where $\omega_C(m,\mathbf{q})$ is the cavity mode frequency, and $G = \frac{i\hbar\Omega_R}{2}$ is the light-matter coupling constant. These equations can be used to find the polariton frequencies as the solutions to

$$[\omega^2(k) - \Omega_M^2][\omega^2(k) - \omega_C^2(m,q)] - \Omega_R^2\omega_C^2(m,q) = 0. \tag{46}$$

Specifically, the polariton frequencies $\omega_\pm(k)$ are given by

$$\omega_\pm^2(k) = \frac{1}{2}\left[\omega_C^2(m,q) + \omega_M^2 + \Omega_R^2 \pm \sqrt{[\omega_M^2 + \Omega_R^2 - \omega_C^2(m,q)]^2 + 4\omega_C^2(m,q)\Omega_R^2}\right]. \tag{47}$$

Substituting the expressions for x_k and z_k into Eq. 41, we get:

$$y_k = \frac{[\omega(k) - \omega_C(m,q)][\omega^2(k) - \Omega_M^2]}{2\sqrt{\omega(k)\omega_C(m,q)}\sqrt{[\omega^2(k) - \Omega_M^2]^2 + \Omega_R^2\omega_C^2(m,q)}}, \tag{48}$$

$$x_k = \frac{[\omega(k) + \omega_C(m,q)][\omega^2(k) - \Omega_M^2]}{2\sqrt{\omega(k)\omega_C(m,q)}\sqrt{[\omega^2(k) - \Omega_M^2]^2 + \Omega_R^2\omega_C^2(m,q)}}. \tag{49}$$

We can rewrite the photon and the matter field operators in terms of the polaritonic operators

$$a_k = x_k^* \Pi_k - y_k \Pi_{-k}^\dagger, \tag{50}$$

$$p_k = z_k^* \Pi_k - t_k \Pi_{-k}^\dagger. \tag{51}$$

The photonic part of each polariton mode $k = (m, \mathbf{q}, \lambda)$ with frequency ω is given by $P_C(\omega) = |x_k|^2 - |y_k|^2$ and matter part is $P_M(\omega) = |z_k|^2 - |t_k|^2$.[24] In terms of the matter and photon frequencies and the collective light-matter coupling strength, $P_C(\omega)$ is given by

$$P_C(\omega) = \frac{(\omega^2 - \omega_M^2 - \Omega_R^2)^2}{(\omega^2 - \omega_M^2 - \Omega_R^2)^2 + \omega_C^2(\omega)\Omega_R^2}, \tag{52}$$

where $\omega_C(\omega)$ is the frequency of the photon mode that hybridizes with matter polarization to give a polariton mode with frequency ω (Eq. 47). The group velocity $v_g(\omega, q)$ of a polariton with frequency ω and in-plane wave vector magnitude q can be obtained by taking the derivative of Eq. 47 with respect to q

$$v_g(\omega, q) = \frac{d\omega_\pm}{dq} = \frac{qc^2}{2\omega}\left(1 \pm \frac{\omega_C^2 - \omega_M^2 + \Omega_R^2}{\sqrt{[\omega_C^2 + \omega_M^2 + \Omega_R^2]^2 - 4\omega_C^2(\omega)\omega_M^2}}\right), \tag{53}$$

where we take the positive sign if the polariton mode with frequency ω is in a UP branch [if $\omega > \omega_C(\omega)$] and the negative sign otherwise.

The previous results will be employed to obtain the polariton density of states $D(\omega)$ defined as

$$D(\omega) = \frac{1}{V} \sum_{\mathbf{q}, m, \lambda, \alpha = \pm} \delta[\omega - \omega_\alpha(m, q)]. \tag{54}$$

We obtain a closed-form expression of the polariton density of states by using

$$\delta[\omega - \omega_\alpha(m, q)] = \frac{\delta[q - q(m, \omega_\alpha)]}{v_g[\omega, q(m, \omega)]} \tag{55}$$

where $q(m,\omega)$ is the in-plane wave vector magnitude that corresponds to the polariton frequency $\omega = \omega_\alpha(m,q)$ for a given mode order m and polarization λ. Substituting this result into Eq. 54 and integrating over the space of in-plane wave vectors, we obtain

$$D(\omega) = \frac{1}{L_C}\sum_{m,\lambda}\frac{q(m,\omega)}{2\pi}\frac{1}{v_g(\omega,q)}\theta[\omega_C(\omega) - m\pi c/L_C] \tag{56}$$

where $\theta[.]$ is the Heaviside step function.

The rate of spontaneous emission of a material system under weak coupling with the electromagnetic field of a polaritonic device is controlled by the photon-weighted polariton DOS (PDOS)

$$D_P(\omega) = P_C(\omega)D(\omega) \tag{57}$$

$$= \frac{1}{L_C}\sum_{m,\lambda}\frac{q(m,\omega)P_C(\omega)}{2\pi v_g(\omega,q)}\theta[\omega_C(\omega) - m\pi c/L_C]. \tag{58}$$

This quantity can be split into the sum of a contribution from $m = 0$ and $m > 0$. For $m = 0$, there is only TM_0 mode contribution, but for $m > 0$, there are both TE and TM modes, resulting in a factor of 2 for the polarization sum. It follows that the PDOS can be written as:

$$D_P(\omega) = \frac{1}{L_C}\sum_{m>0}\frac{q(m,\omega)P_C(\omega)}{\pi v_g(\omega,q)}\theta[\omega_\mathrm{C} - m\pi c/L] + \frac{q_0(\omega)P_C(\omega)}{2\pi L_C v_g[\omega, q_0(\omega)]}, \tag{59}$$

where $q_0(\omega) = q(m = 0,\omega)$. This expression for $D_P(\omega)$ can be written as a single sum by denoting polariton frequencies by ω and writing

$$D_P(\omega) = \sum_{m=0}^{\infty}\left(1 - \frac{\delta_{0,m}}{2}\right)\frac{q(m,\omega)P_C(\omega)}{\pi v_g[\omega, q(m,\omega)]L_C}\theta[\omega_\mathrm{C}(\omega) - m\pi c/L_C]. \tag{60}$$

The thermal radiation energy density $\rho_P(\omega,T)$ in a polaritonic system can be written as the product of the polariton energy, the Bose-Einstein distribution, and the photon-weighted

polariton density of states

$$\rho_P(\omega, T) = D_P(\omega) \frac{\hbar\omega}{e^{\frac{\hbar\omega}{k_B T}} - 1}. \tag{61}$$

Substituting the expression for $D_P(\omega)$, we obtain:

$$\rho_P(\omega, T) = \sum_{m=0}^{\infty} \left(1 - \frac{\delta_{0,m}}{2}\right) \frac{q(m,\omega) P_C(\omega)}{\pi v_g[\omega, q(m,\omega)] L_C} \frac{\hbar\omega}{e^{\frac{\hbar\omega}{k_B T}} - 1} \theta[\omega_{\mathrm{C}}(\omega) - m\pi c/L_C]. \tag{62}$$

This expression can be used to calculate the contribution of polaritonic modes to the overall thermal radiation inside a microcavity, considering both the upper and lower polariton branches. Equation 62 is particularly useful for computing the thermal absorption and emission rates of a charged system weakly coupled to a polaritonic material.

7 HF and LiH molecules

In Fig. S4, BIRD rates in the weak coupling regime are shown relative to the free-space rate. For small microcavity lengths ($L_C < 2$ μm for HF and $L_C < 5$ μm for LiH), a mild but consistent enhancement of BIRD rates is observed due to an increased microcavity electromagnetic DOS over a wide frequency range. As L_C increases, the microcavity BIRD rates converge to the free-space rates.

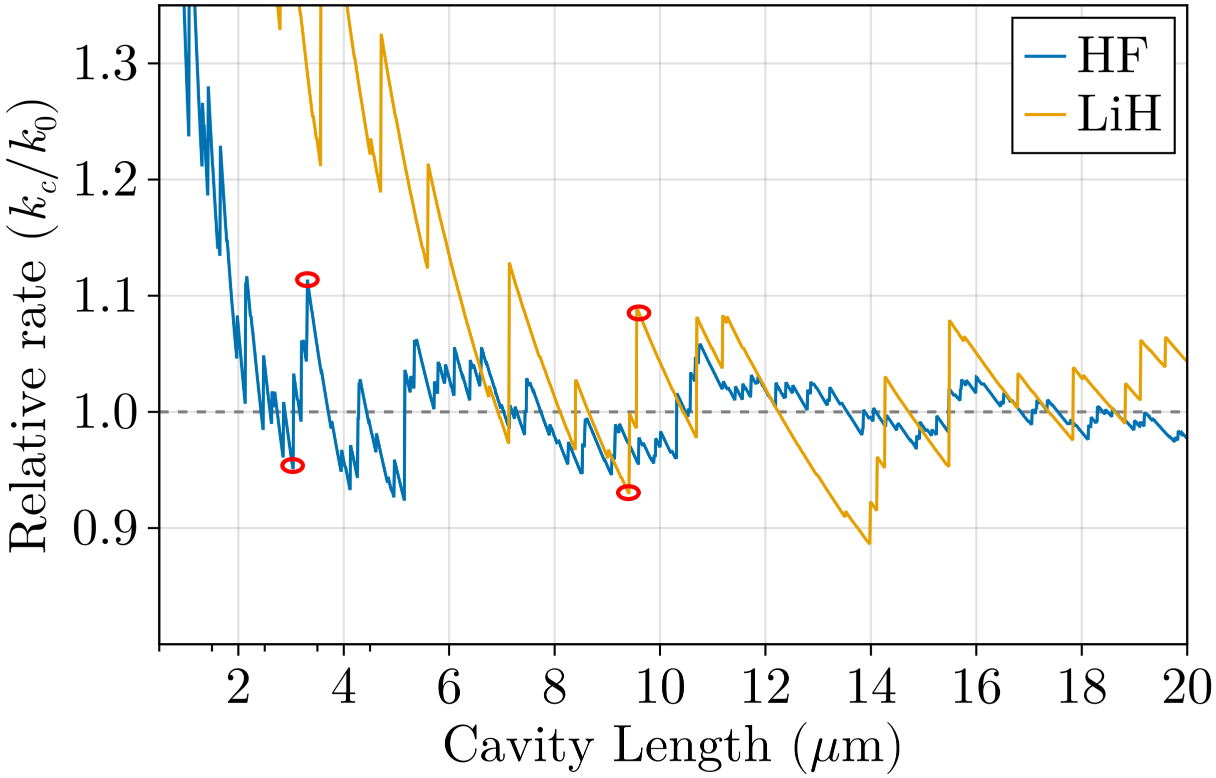

Figure S4: Ratio of BIRD rate inside a microcavity (weak coupling regime) to the free-space rate as a function of microcavity length for HF and LiH molecules. Red circles highlight specific lengths further discussed in Fig. S5.

The oscillatory behavior in Fig. S4 arises from discrete vibrational transitions being enhanced or suppressed depending on L_C. Overtones play an essential role, with HF dissociation being most influenced by transitions $i \to 23$, with $i \in 16, 17, 19$, and LiH by transitions $i \to 29$, with $i \in 21, 22, 23, 25$. These overtones excite the system to the highest energy-bound

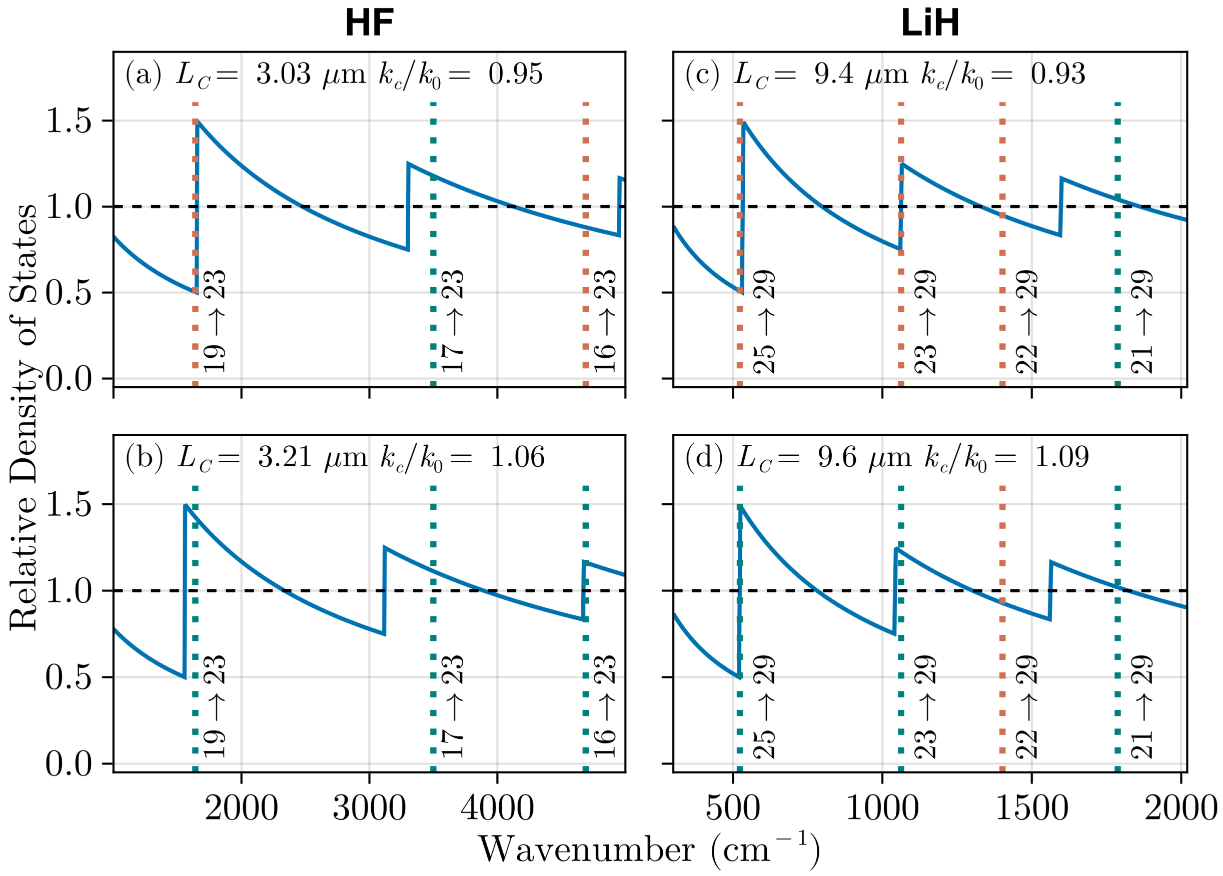

Figure S5: Microcavity photon DOS normalized to the free space DOS for selected lengths (L_C) where BIRD suppression and enhancement were observed for HF (left panels) and LiH (right panels) in Fig. S4. The dotted vertical lines indicate the location of the transition frequencies corresponding to the most relevant overtone transitions, with color coding to distinguish between suppression (red) and enhancement (green). Panel (a) shows that the small HF BIRD suppression observed at $L_C = 3.03$ μm is at least in part caused by the reduced relative DOS at the highlighted overtones. Panel (b) shows the mild enhancement of HF BIRD in a microcavity with $L_C = 3.21$ μm can be ascribed to the greater microcavity photon DOS at the frequencies of the highlighted overtones. Panels (c) and (d) show the analogous scenario for LiH, where BIRD is observed to be suppressed at $L_C = 9.4$ μm but enhanced at $L_C = 9.6$ μm, primarily due to the variation of the microcavity photon density of states at the selected $i \to 29$ overtone frequencies.

states, leading to dissociation. Fundamental transitions ($i \to i+1$) at high excitation levels involve small energies, leading to reduced photon populations and oscillator strengths.

Fig. S5 presents the microcavity DOS normalized to free space for selected L_C values where BIRD suppression or enhancement was observed. The suppression at $L_C = 3.03$ μm for HF and $L_C = 9.4$ μm for LiH correlates with suppressed overtones, while enhancement at $L_C = 3.21$ μm (HF) and $L_C = 9.6$ μm (LiH) corresponds to enhanced microcavity photon DOS at relevant overtone frequencies. Finite linewidths in radiative transitions would smooth oscillations and dampen their amplitude, limiting microcavity effects to small L_C.

BIRD suppression effects remain minor as multiple dissociative pathways exist. Blocking a single transition does not significantly impact dissociation, reinforcing two key findings: (i) overtone transitions are crucial, and (ii) blocking a single reactive pathway is largely inconsequential.

Fig. S6 shows polariton-assisted BIRD rates as a function of host matter frequency (ω_M) for HF ($L_C = 3.21$ μm, $\Omega_R = 400$ cm^{-1}) and LiH ($L_C = 9.6$ μm, $\Omega_R = 200$ cm^{-1}). Enhancement peaks occur when ω_M is slightly above a diatomic overtone frequency ($i = 17 \to 23$ for HF, $i = 22 \to 29$ for LiH). Suppression occurs when ω_M approaches these transitions from the left, placing them in the stopgap region where the electromagnetic DOS is zero.

Fig. S7 shows the photon-weighted polariton DOS $D_P(\omega)$ for selected Ω_R at $L_C = 9.6$ μm and $\omega_M = 1401$ cm^{-1}. Strong coupling alters $D_P(\omega)$ near ω_M, influencing vibrational transition rates. The singularity in $D_P(\omega)$ allows a transition to become arbitrarily fast compared to free space, but reaction rates remain finite due to other limiting steps.

Experimentally, imperfections in the electromagnetic device and material disorder smooth out $D_P(\omega)$ near the stopgap, reducing BIRD enhancement compared to theoretical predictions. Including finite linewidths relaxes strict resonance conditions, making enhancement dependent on the spectral overlap between $\omega_{i\to j}$ and $D_P(\omega)$. Thus, reported results represent upper bounds for polariton-assisted BIRD rates under ideal conditions.

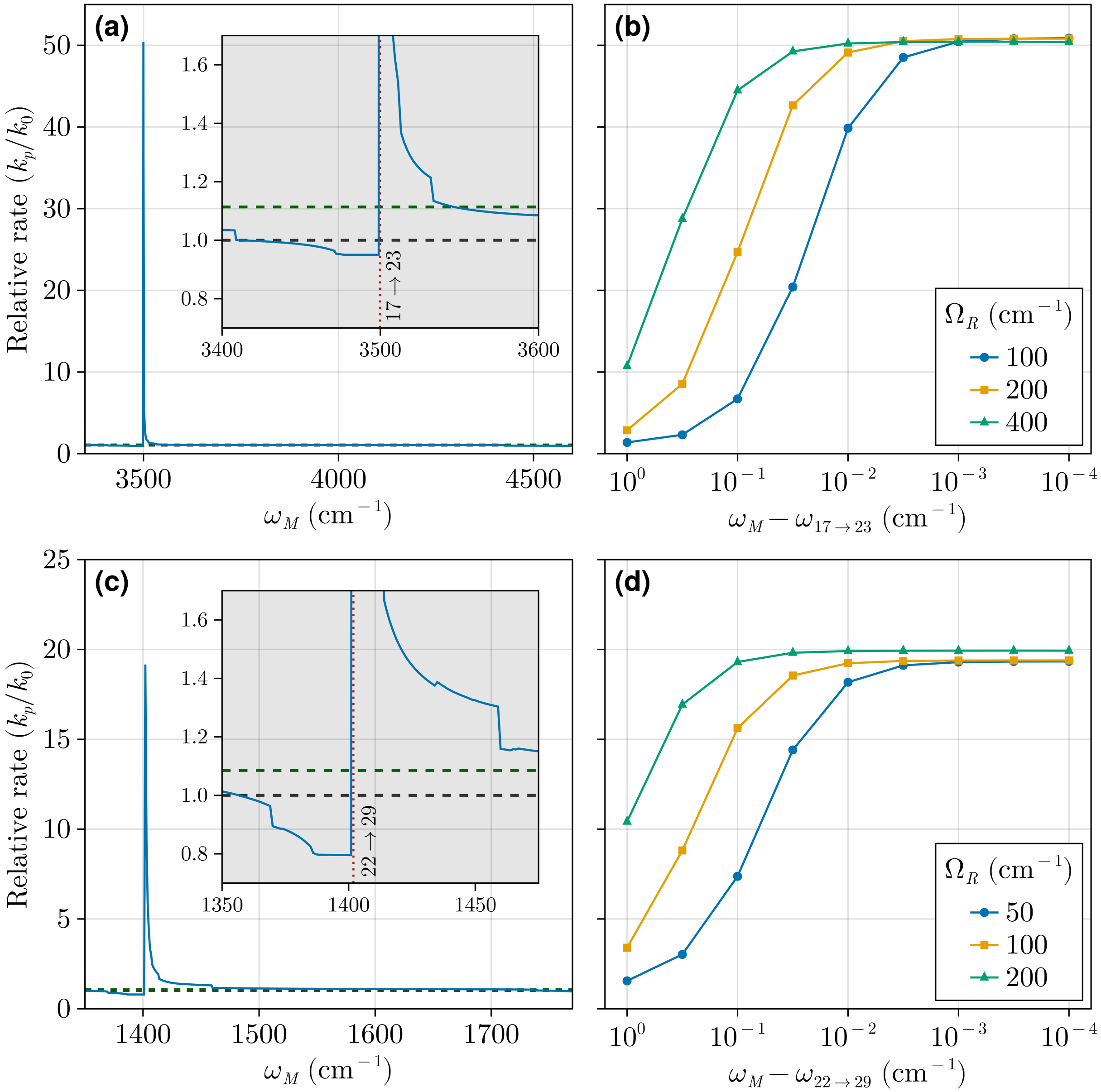

Figure S6: Ratio of polariton-assisted BIRD rates to free space BIRD rates. In the polaritonic case, the diatomic molecule is embedded in a strongly coupled microcavity with variable host material with frequency ω_M. The top panels, (a) and (b), correspond to HF, and the bottom panels, (c) and (d), show results for LiH. In (a) and (c), the collective light-matter interaction strength, Ω_R, is fixed at 400 and 200 cm^{-1}, respectively. The horizontal dashed gray and green lines indicate where the dissociation rates equal k_0 and k_c, respectively. The insets in (a) and (c) show zoomed-in views around the overtones $17 \to 23$ for HF and $22 \to 29$ for LiH. In (b) and (d), relative BIRD rates are shown for different Rabi frequencies (Ω_R), depicting the dependency on the detuning between the host molecule and the diatomic transition energies.

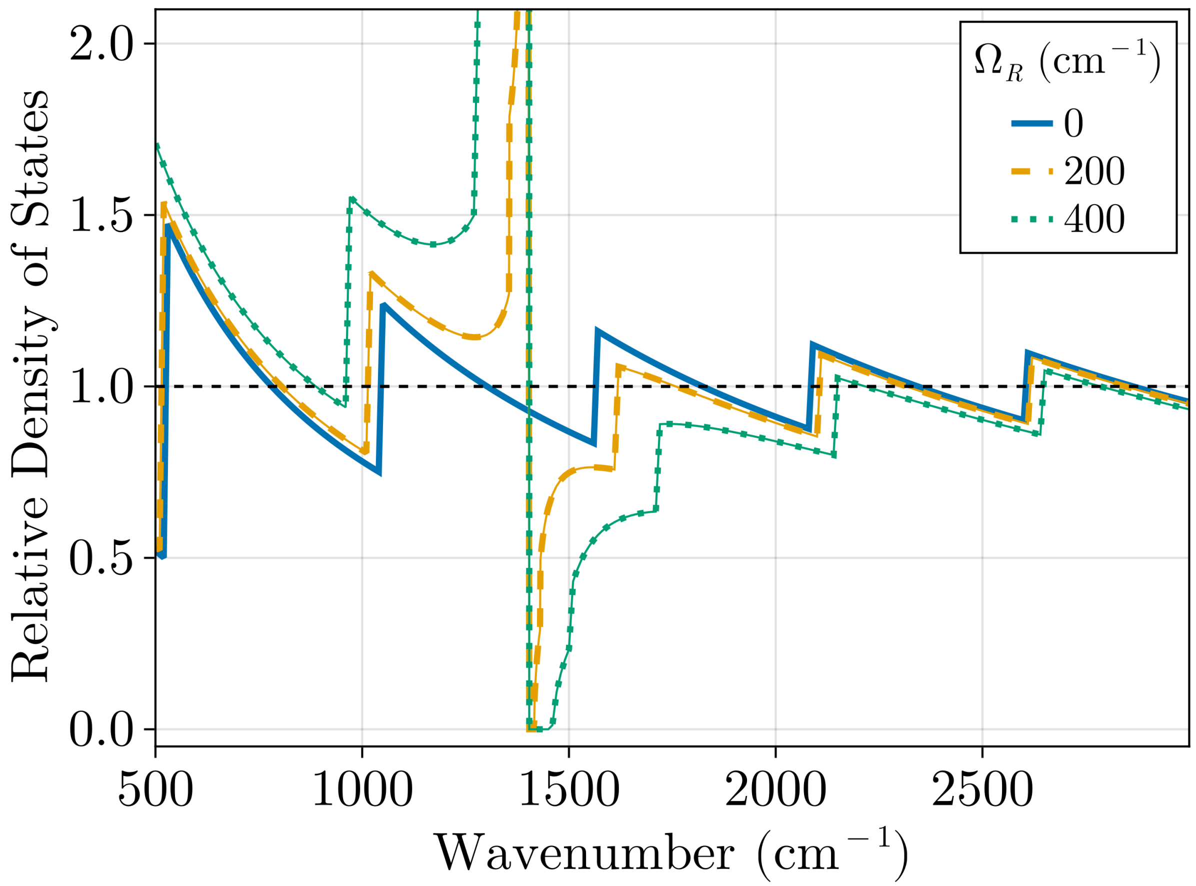

Figure S7: Photon-weighted polariton density of states $D_P(\omega)$, normalized to the free space DOS, for a microcavity with length $L_C = 9.6$ μm strongly coupled to a material with a bright transition at frequency $\omega_M = 1401$ cm^{-1}. The curves show the effect of different Rabi splitting values (Ω_R) on the photon-weighted polariton DOS, which is relevant for radiative processes mediated by polaritonic systems. As Ω_R increases, the frequency range below ω_M, where the photon-weighted polariton DOS significantly exceeds that of free space, widens. This trend explains the impact of the collective light-matter interaction strength on the observed polariton-assisted bond infrared dissociation (BIRD) enhancement shown in Fig. S6.

8 Dissociation Rates in Lossy Cavities

The preceding analysis assumed ideal optical microcavities with perfectly reflecting mirrors. In practice, the mirrors are either metallic or multilayer dielectric stacks with alternating refractive indices, known as distributed Bragg reflectors (DBRs).[25] Both devices are imperfect and inevitably introduce losses: finite conductivity limits the reflectivity of metallic mirrors and causes ohmic absorption, whereas DBRs suffer from material absorption and fabrication imperfections. In this section we evaluate how such losses, focusing on weakly coupled microcavities with absorptive metallic mirrors, affect the corresponding BIRD rates obtained assuming perfect mirrors (Sec. III. A). We also provide comments on the polariton-assisted case.

8.1 Computational Method.

In microcavities with lossy mirrors, the Green function formalism described e.g., in Ref.[18] can be adopted to obtain the local electromagnetic density of states in terms of the imaginary part of the electromagnetic Green tensor.

Following Ref.,[18] the photon LDOS relative to free space at position z_0 inside a planar microcavity consisting of identical front and back metallic mirrors separated by a distance L is given by

$$\frac{D_C(\omega, z_0)}{D_0(\omega)} = 1 + \frac{1}{2}\mathrm{Re}\int_0^{\infty} \frac{u}{\sqrt{1-u^2}} \left[\frac{N_{\mathrm{TE}}(u)}{D_{\mathrm{TE}}(u)} + \frac{N_{\mathrm{TM}}(u)}{D_{\mathrm{TM}}(u)}\right] du, \tag{63}$$

where $u = \sin\theta$ is the in-plane propagation angle, and N_{TE}, N_{TM}, D_{TE}, and D_{TM} are related

to TE and TM reflection coefficients r_{TE} and r_{TM} according to

$$N_{\text{TE}} = r_{\text{TE}} e^{2ik_z z_0} + r_{\text{TE}} e^{2ik_z (L-z_0)} + 2r_{\text{TE}}^2 e^{2ik_z L}, \tag{64}$$

$$D_{\text{TE}} = 1 - r_{\text{TE}}^2 e^{2ik_z L}, \tag{65}$$

$$N_{\text{TM}} = (2u^2-1) r_{\text{TM}} (e^{2ik_z z_0} + e^{2ik_z (L-z_0)}) + 2r_{\text{TM}}^2 e^{2ik_z L}, \tag{66}$$

$$D_{\text{TM}} = 1 - r_{\text{TM}}^2 e^{2ik_z L}, \tag{67}$$

where $k_z = \sqrt{\epsilon_1}\omega/c\sqrt{1-u^2}$ is the transverse component of the wave vector inside the microcavity, and ϵ_1 is the dielectric constant of the background medium (taken as 1 for vacuum). The complex-valued Fresnel reflection amplitudes r_{TE} and r_{TM} are evaluated using the dielectric function of the mirrors here approximated by the Drude form

$$\epsilon_2(\omega) = 1 + \frac{\omega_p^2}{\omega^2 + i\gamma\omega}, \tag{68}$$

where ϵ_2 is the metal complex dielectric function, ω_p is the plasma frequency and γ is the damping rate.

Table S3 below presents the Drude parameters employed for each metal considered in our study. All parameters were selected based on experimentally reported values corresponding to optimal film thicknesses that maximize plasmonic thermal conductivity.[26]

Table S3: Drude parameters used for dielectric function calculations.

Material	ω_p (rad/s)	γ (rad/s)
Au	1.22×10^{16}	7.11×10^{13}
Al	1.82×10^{16}	1.23×10^{14}
Pt	7.87×10^{15}	1.22×10^{14}

In our radiative transition rate computations, we employ the isotropically averaged LDOS spatially averaged over all emitter positions inside the microcavity to account for spatial and angular variation of the field distribution inside the cavity and the motion of the diatomic which is appreciable in the macroscopic timescales required for BIRD to happen at moderate

temperatures. In this case, the relative LDOS modulates the spontaneous emission rate according to

$$\Gamma_{\rm sp}^{\rm C}(\omega) = \Gamma_{\rm sp}^{0}(\omega)\frac{D_C(\omega)}{D_0(\omega)}, \tag{69}$$

where $\Gamma_{\rm sp}^{\rm C}(\omega)$ and $\Gamma_{\rm sp}^{(0)}$ are dipolar microcavity and free space spontaneous emission rates corresponding to a quantum level transition with frequency ω. Using Einstein coefficient relations described in the main text, the stimulated emission and absorption rates can then be obtained from spontaneous emission rates and the Pauli Master equation employed for BIRD can be implemented as described in the main manuscript.

Prior to examining the effect of microcavity losses on BIRD rates, we present in Fig. S8 simulations of the microcavity density of states (normalized by the free-space photon DOS) for a cavity length (L = 25) m. These calculations employ metallic mirrors composed of Au, Al, and Pt using Fresnel reflection coefficients derived from the Drude model as described above.

As shown in Figure S8, the ideal perfect mirror microcavity exhibits sharp, periodic resonances corresponding to discrete photonic modes. In contrast, realistic metallic mirrors introduce damping and spectral broadening due to absorption losses, resulting in smoother density of states profiles. Notably, both Au and Al demonstrate enhanced local density of optical states at low frequencies despite their lossy characteristics. This enhancement arises from evanescent field contributions near the metal interfaces, where dissipative surface modes can significantly increase the local electromagnetic energy density. While these evanescent components exhibit limited propagation into the resonator interior, their presence near the interfaces enhances the average DOS experienced by molecules in close proximity, particularly in resonators of short length. Consistent with the $1/\sqrt{\omega}$ frequency dependence of the evanescent decay length, a gradual approach to the free-space DOS limit is observed at higher frequencies.

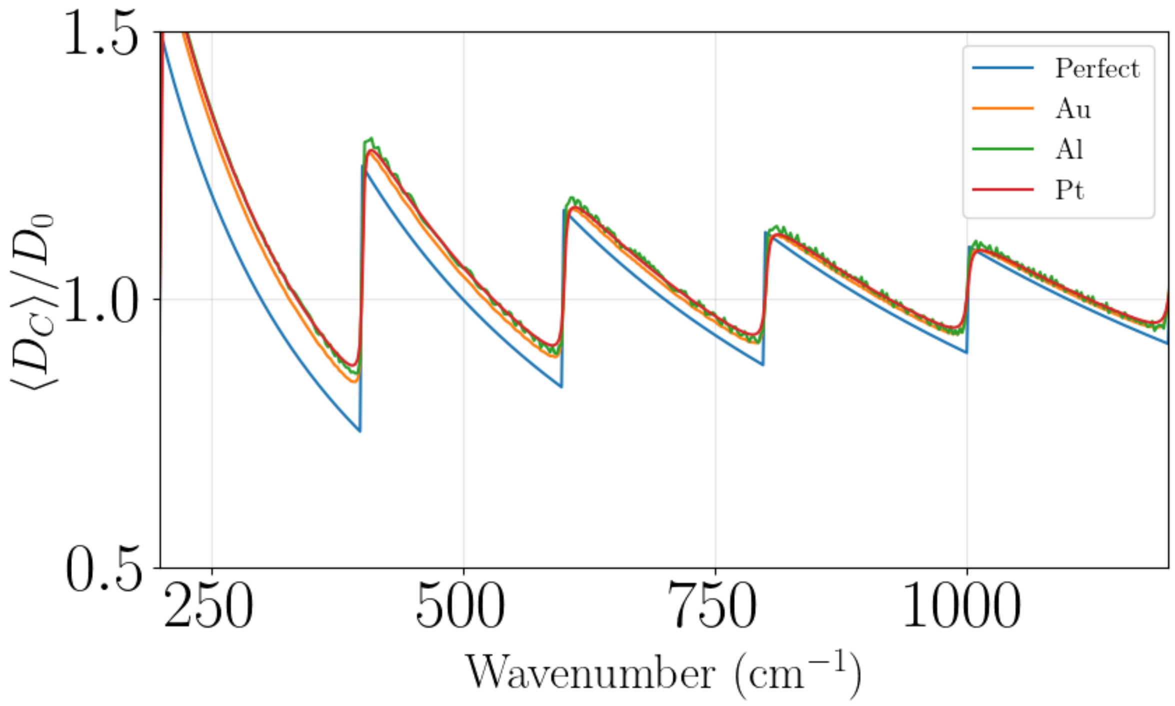

Figure S8: Isotropically and spatially averaged LDOS $\langle D_C \rangle_{z_0}/D_0$ at $L = 25$ μm for perfect mirrors and three metal mirrors (Au, Al, Pt). Realistic metals suppress and broaden the modal features seen in the ideal case.

8.2 Results

Figure S9 compares the BIRD dissociation rates relative to free space k_c/k_0 as a function of microcavity length for perfect mirrors (as in our manuscript) and lossy mirrors made from gold and aluminum.

Several key inferences can be made from Fig. S9. First, the introduction of imperfect absorptive metallic mirrors yields relative BIRD rate enhancements of the same order of magnitude as microcavities with perfect mirrors. Second, simulations with lossy mirrors broaden the sharp resonance features observed in the perfect microcavity length-dependent BIRD rate variation.

Interestingly, at short microcavity lengths, both metallic mirror types exhibit enhanced BIRD rates relative to perfectly reflecting mirrors. This enhancement can be attributed to evanescent electromagnetic waves supported at the metal–dielectric interfaces,[27,28] as demon-

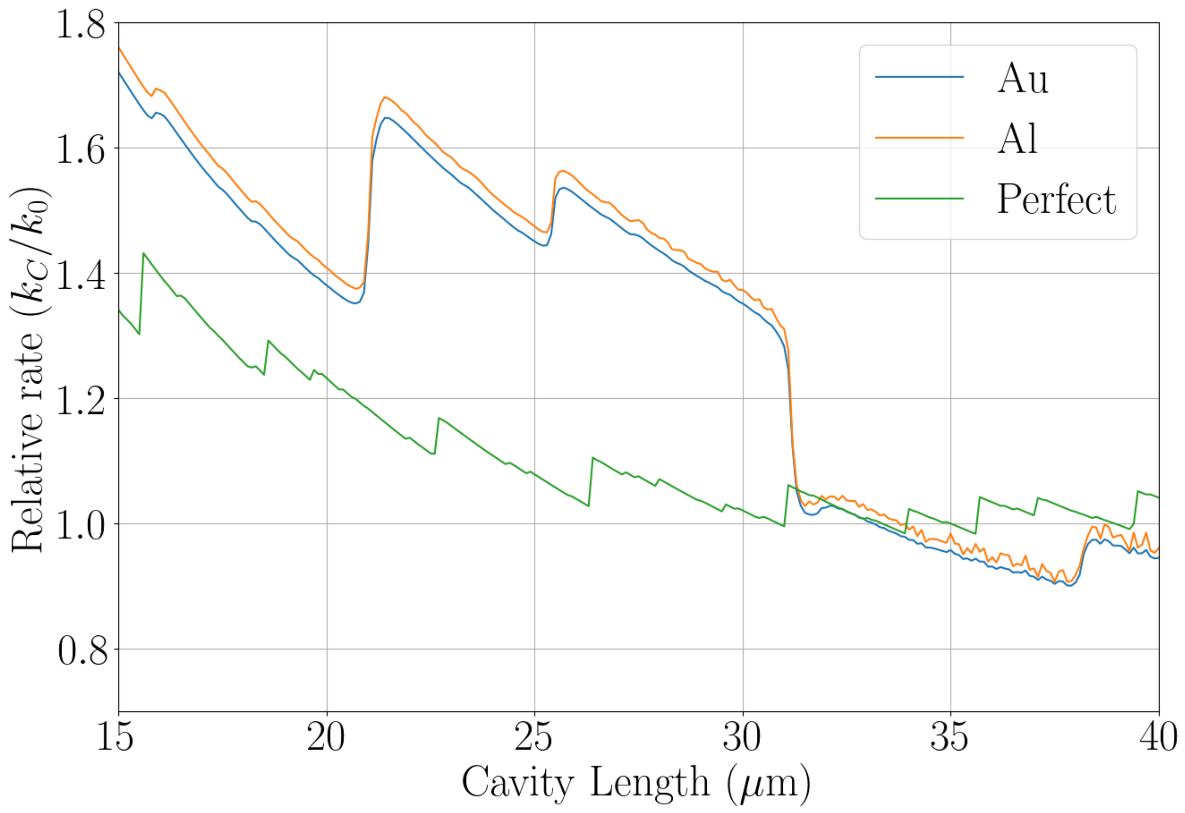

Figure S9: Relative BIRD rate k_c/k_0 versus cavity length L at $T = 400$ K. Mirror materials include gold (Au), aluminum (Al), and an idealized perfect reflector. Lossy mirrors broaden and suppress the resonances, but maintain rate enhancements at shorter lengths.

strated in Fig. S8. In lossy resonators, these near-field electromagnetic field components increase the accessible electromagnetic energy density for molecular transitions, leading to enhanced reaction rates. Although these modes are non-propagating and decay exponentially from the interface, their influence remains significant in confined geometries.[28] Aluminum mirrors produce slightly higher enhancements due to their marginally greater penetration depth at infrared frequencies.[29] As the resonator length increases, the contribution of evanescent modes diminishes due to their finite penetration length, and the field intensity depleting absorptive losses imparted by the mirrors become of greater significance. This leads to the weak suppression observed at microcavity lengths greater than 30 μm. Nevertheless, as L increases, all examined scenarios show slow weak converge toward the free-space dissociation rate limit, as illustrated in Fig.S9.

In summary, incorporating realistic mirror losses reveals that microcavity LDOS and BIRD dissociation rate enhancements persist at short microcavity lengths. The enhancement magnitude depends on the mirror material properties. While our model primarily considers radiative contributions, we acknowledge that evanescent near-field modes supported by metallic surfaces may also contribute to the observed LDOS enhancements. Their spatially localized nature and coupling to molecular dipoles can enhance transition rates even in the presence of loss. Nevertheless, the order of magnitude of BIRD rate modifications remains consistent between perfect and imperfect microcavities.

8.3 Comment about polariton-assisted mechanism in lossy resonators

The treatment of lossy mirror effects introduced above was applied specifically to the empty microcavity case described in Section 3A of the main text. These findings strongly suggest that realistic metallic mirrors would influence polariton-assisted rates in the same qualitative manner: sharp spectral features would become smoother and broader, yet the order-of-magnitude BIRD enhancements predicted for microcavities with perfect mirrors are very likely to persist as they do in the weak coupling analysis. This is expected, especially as, our polariton-assisted simulations already incorporate an effective light-matter coupling cutoff that eliminates the highly off-resonant couplings responsible for the stop gap singularity observed in the polariton-weighted photon density of states.

Our introduction of a high energy cutoff effectively removes the polariton stop gap singularity from consideration. Therefore, incorporating microcavity losses is not expected to alter our conclusions regarding the order of magnitude of the observed polariton-assisted rate enhancement. Nevertheless, we recognize that a comprehensive treatment of microcavity losses in the strong coupling regime represents a relevant and important consideration for future investigations.

9 Sensitivity Analysis

Due to the modified DOS, microcavities may enhance or suppress multiple vibrational transitions (relative to free space) in both weak and strong coupling regimes. We performed a sensitivity analysis to find which transitions are the most impactful to the BIRD rate. This consists of the following procedure for each fundamental or overtone transition frequency ω_{ij}.

We perturb the free space photon DOS at ω_{ij} by scaling this quantity by a factor of δ

$$D_0(\omega_{ij}) \to \delta \cdot D_0(\omega_{ij}). \tag{70}$$

Since transition rates are proportional to the DOS, the transitions associated with the absorption or emission of photons with frequency ω_{ij} are effectively scaled by the same factor, while all other transitions remain with free space rates. The corresponding perturbed transition matrix, $\mathbf{J}$ is then constructed, and from its lowest eigenvalue, a perturbed BIRD rate is obtained and assigned a sensitivity score S_{ij} based on the definition

$$S_{ij} = \frac{\text{free space BIRD rate after perturbation}}{\text{free space BIRD rate with no perturbation}} - 1\,. \tag{71}$$

Repeating this procedure for all $i \neq j$ combinations yields a sensitivity matrix $\mathbf{S}$ shown as a heat map in Fig. S10. The S matrix is symmetric given that emission and absorption depend on the photon DOS at the same frequency, i.e., it is impossible to enhance/suppress one process without also affecting the reverse process.

From Fig. S10, we see that the most important transitions are overtones that reach the final bound state from relatively high energy vibrational levels ($i \to 54$). We list these transitions, along with their energies and sensitivity scores in Table S4. The fact that these overtones dominate the dissociation dynamics may be understood from the observation that, in anharmonic systems, fundamental transitions $i \to i \pm 1$ are increasingly slowed as

we climb the energy level ladder. For example, fundamental transitions starting at $n = 40$ involve energies under 50 cm^{-1}. Such transitions have, in general, weak oscillator strength and correspond to small DOS and thermal photon populations. Hence, overtones play a dominant role at these higher energy levels and act as bottlenecks for dissociation from the highest energy-bound state.

Table S4: Most important transitions according to their sensitivity analysis score for NaLi.

**Transition**	**Energy (cm^{-1})**	**Sensitivity** ($\delta = 1.5$)	**Sensitivity** ($\delta = 0.5$)
$42 \rightarrow 54$	379.8	0.072	-0.073
$43 \rightarrow 54$	322.6	0.065	-0.066
$48 \rightarrow 54$	106.1	0.054	-0.055
$49 \rightarrow 54$	76.8	0.049	-0.05
$41 \rightarrow 54$	441.7	0.048	-0.048
$47 \rightarrow 54$	140.1	0.036	-0.036
$44 \rightarrow 54$	270.0	0.031	-0.031
$50 \rightarrow 54$	52.1	0.029	-0.029
$40 \rightarrow 54$	508.3	0.017	-0.017
$51 \rightarrow 54$	32.1	0.011	-0.012

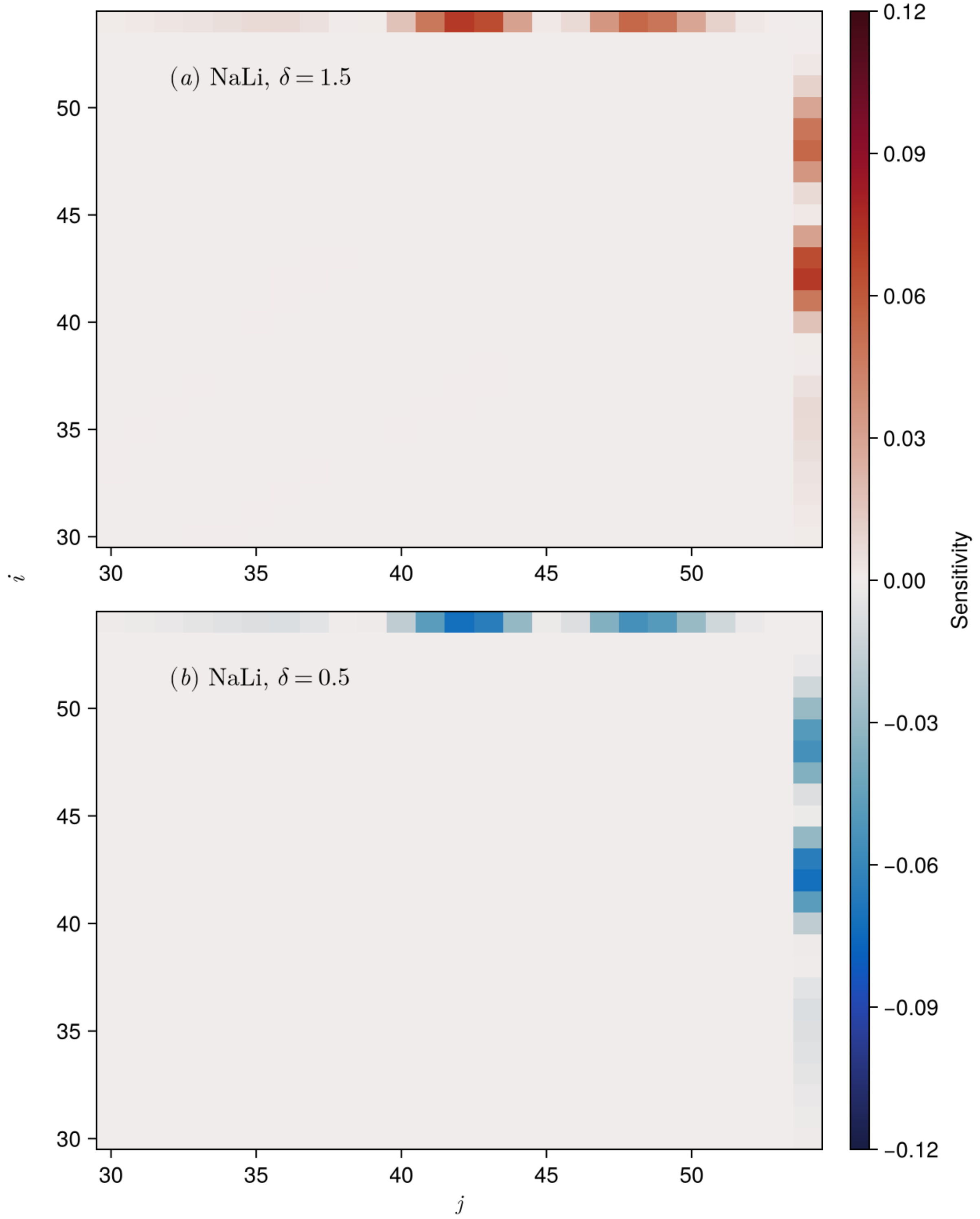

Figure S10: Sensitivity analysis for NaLi under enhancement ($\delta = 1.5$) and suppression ($\delta = 0.5$). The sensitivity was calculated using Eq. 71 following the procedure described in this section. Transitions not shown in the plot have negligible sensitivity.

10 Temperature Dependence

Diatomic molecules such as HF and LiH are bound by a strong covalent bond that is stable even at temperatures much higher than room temperature. This means their dissociation from the most energetic bound state by thermal radiation can only happen at very high temperatures. In this section, we discuss the temperature dependence of the reported relative rates and demonstrate that the qualitative analysis presented in the main manuscript holds for all examined temperatures.

Achieving numerically stable BIRD rate constants (lowest eigenvalue of transition matrix **J**) is straightforward at sufficiently high temperatures (e.g., comparable to or larger than the vibrational temperatures of the modeled diatomics). At such temperatures (e.g., 2000 K for LiH and 4000 K HF), the rate constants for absorption and emission are large enough that floating point arithmetic errors are negligible. In Fig. S11, we present relative BIRD rates for temperatures as low as 1000 K in weak and strong coupling regimes. To mitigate floating point errors, these computations were performed using quadruple precision (FLOAT128).

In previous sections of this SI, we explain that a small change in the cavity length (9.4 to 9.6 μm) leads to a measurable change in the LiH dissociation rate (k_c/k_0 shifts from 0.93 to 1.09) - the same qualitative result is also present for NaLi in the main text. In Fig. S11(a), this observation remains valid for a large range of temperatures. Although at lower temperatures the enhancement (at $L_C = 9.6\,\mu$m) is smaller, the suppression effect ($L_C = 9.4\,\mu$m) is virtually temperature-independent. Moreover, the sudden change in k_c/k_0 between the two examined cavity lengths is still present for all temperatures.

Relative BIRD rates for LiH in the strong coupling regime are shown in Fig. S11(b) for a range of temperatures. Here we present results for two specific values of matter host frequency: $\omega_M = 1399$ cm^{-1}, which causes the $22 \to 29$ overtone to be completely suppressed and $\omega_M = 1402$ cm^{-1} where the $22 \to 29$ overtone transition rate is greatly enhanced leading to a increased BIRD rate. From Fig. S11 (b), we observe a moderate temperature dependence when $\omega_M = 1402$ cm^{-1}. However, the qualitative picture remains the same: when $\omega_M = 1399$

cm^{-1} , the suppression of the 22 → 29 overtone causes a mild reduction of the BIRD rate, whereas at $\omega_M = 1402$ cm^{-1}, this overtone transition rate is enhanced and polariton-assisted BIRD via the highest energy bound state is faster by one order of magnitude relative to free space.

Selected numerical values of k_0, k_c, and k_p are shown in Table S5. Although polariton-assisted enhancements (k_p/k_0) become more significant at lower temperatures, Table S5 shows BIRD rates are extremely low at the smallest examined temperatures. For example, at T = 1000 K k_0 is on the order of $O(10^{-12}/s)$. Hence, we focus our analysis on temperatures comparable to the LiH and HF vibrational temperatures. While a more comprehensive examination of thermal effects is left for future work, in the next section we explore a simple toy model to gain insight into the origin of this temperature dependence on polariton enhancements.

Table S5: Absolute BIRD rates for LiH in free space (k_0), weak coupling regime (k_c), and strong coupling regime (k_p). In all cases, $L_C = 9.6$ μm, $\Omega_R = 200$ cm^{-1}, and $\omega_M = 1402$ cm^{-1}.

**Temperature (K)**	k_0 (s^{-1})	k_c (s^{-1})	k_p (s^{-1})
1000	5.96×10^{-12}	6.39×10^{-12}	3.26×10^{-10}
1500	1.18×10^{-7}	1.27×10^{-7}	3.33×10^{-6}
2000	1.64×10^{-5}	1.78×10^{-5}	3.14×10^{-4}
2500	3.15×10^{-4}	3.42×10^{-4}	4.56×10^{-3}
3000	2.23×10^{-3}	2.43×10^{-3}	2.58×10^{-2}

10.1 Temperature Dependence of a Model System

In this section, we examine a minimal BIRD system with very few degrees of freedom that affords an analytical examination of its temperature dependence. The smallest system that can be studied is a Morse oscillator with two bound states [Fig. S12 (a)] with the transport

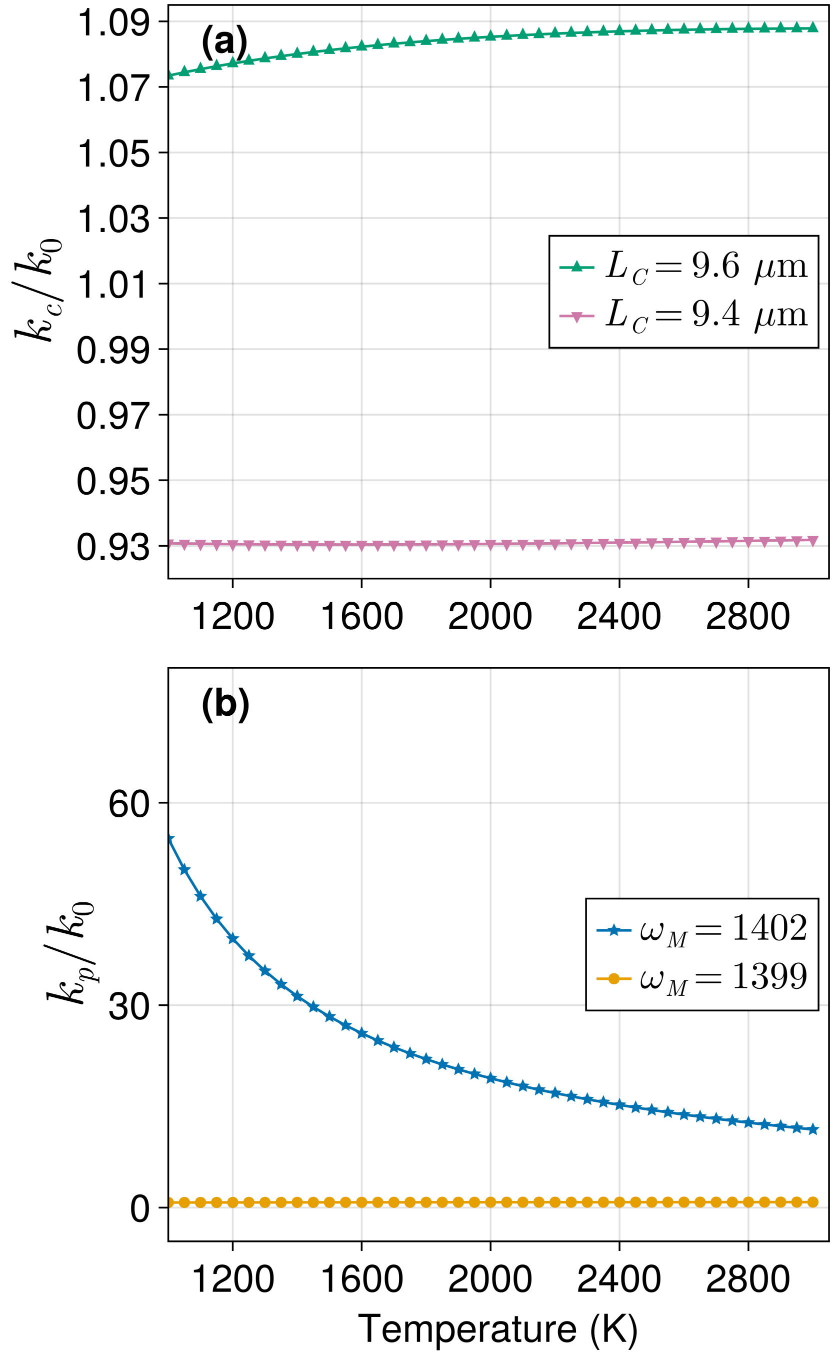

Figure S11: Temperature dependence of the relative BIRD rates for LiH in the **(a)** weak and **(b)** strong coupling regimes. In **(b)**, $\Omega_R = 200$ cm^{-1} and $L_C = 9.6\ \mu$m

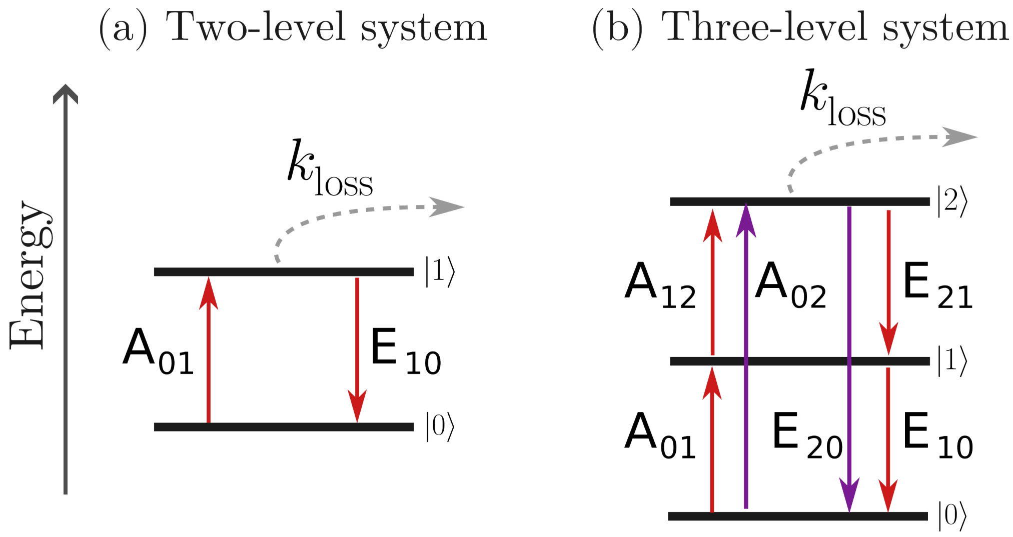

Figure S12: Minimal systems with two and three levels for BIRD rates are analytically examined.

matrix given by

$$\mathbf{J} = \begin{bmatrix} A_{01} & -E_{10} \\ -A_{01} & k_{\rm loss} \end{bmatrix}, \tag{72}$$

where A_{ij} and E_{ij} are the absorption of emission rates from i to j, respectively. Since $k_{\rm loss}$ is much greater than any other rates, the lowest eigenvalue, and therefore BIRD rate is A_{01}. Hence, the temperature dependence of the BIRD rate for this trivial case is given by the Bose-Einstein factor. A more interesting picture emerges when three energy levels are considered, represented in Fig. S12 (b). The transport matrix in this case is written as

$$\mathbf{J} = \begin{bmatrix} A_{01}+A_{02} & -E_{10} & -E_{20} \\ -A_{01} & E_{10}+\alpha A_{12} & -\alpha E_{21} \\ -A_{02} & -\alpha A_{12} & k_{\rm loss} \end{bmatrix}, \tag{73}$$

where the parameters α is introduced here to model the change due to polaritonic enhancement. In general, the polariton-modified density of states will affect multiple transitions if they are close enough to the polariton resonance ω_M. However, for simplicity we will consider

here that only one transition is significantly enhanced and its enhancement is quantified by α. The characteristic polynomial of this matrix is

$$
\begin{aligned}
0 = &(A_{01} + A_{02} - \lambda) \cdot (E_{10} + \alpha A_{12} - \lambda) \cdot k_{\text{loss}} && (74)\\
&- \alpha E_{10} E_{21} A_{01} - \alpha E_{20} A_{01} A_{12} && (75)\\
&- \alpha^2 (A_{01} + A_{02} - \lambda) E_{21} A_{12} - E_{10} A_{01} k_{\text{loss}} && (76)\\
&- (E_{10} + \alpha A_{12} - \lambda) E_{20} A_{02} \, . && (77)
\end{aligned}
$$

Dividing this equation by k_{loss} and recognizing that $k_{\text{loss}} \gg A_{ij},\, E_{ij}\ \forall i, j$, we can discard any terms that do not contain k_{loss}. Thus, we obtain the quadratic equation

$$
\lambda^2 - (A_{01} + A_{02} + E_{10} + \alpha A_{12})\lambda + (A_{01} + A_{02})(E_{10} + \alpha A_{12}) - E_{10} A_{01} = 0 \, . \tag{78}
$$

Since all rates are positive and the lowest eigenvalue of the transport matrix must be positive, λ can be computed as

$$
\lambda = \frac{1}{2} \left(-b - |b| \sqrt{1 - \frac{4ac}{b^2}} \right) , \tag{79}
$$

where $a = 1.0,\ b = -(A_{01} + A_{02} + E_{10} + \alpha A_{12})$, and $c = (A_{01} + A_{02})(E_{10} + \alpha A_{12}) - E_{10} A_{01}$. We are going to mostly be concerned with the behavior of this equation at low temperatures where spontaneous emission is much faster than absorption of stimulated emissions. Hence, $\frac{4ac}{b^2} \ll 1$ and we can use $\sqrt{1 - \frac{4ac}{b^2}} \approx 1 - \frac{2ac}{b^2}$. Eq. 79 becomes

$$
\lambda \approx \frac{ac}{b} = \frac{(A_{01} + A_{02})(E_{10} + \alpha A_{12}) - E_{10} A_{01}}{(A_{01} + A_{02} + E_{10} + \alpha A_{12})} \tag{80}
$$

10.1.1 Low Temperature Free-space limit

In free-space there is no polaritonic enhancement, thus $\alpha = 1.0$. At low temperatures, spontaneous emission is the dominant process. This allow us to simplify the denominator of Eq.

80 as $A_{01}+A_{02}+E_{10}+\alpha A_{12}\approx E_{10}$. A similar simplification is applied to the numerator and we are left with

$$\lambda_1 \approx A_{02}\,. \tag{81}$$

This result can be rationalized as follows: any population in state $|1\rangle$ will be quickly depleted by spontaneous emission before it can move to the doorway state ($|2\rangle$), hence the overtone $0\rightarrow 2$ is the only viable way to reach the final state that reacts rapidly before it can emit back to state $|1\rangle$, because $k_{\text{loss}} \gg E_{21},\ E_{20}$. The validity of this approximation is shown numerically in Fig. X, where we see that the qualitative temperature dependence is well captured and at low enough temperatures there is also a good quantitative agreement.

10.1.2 Low Temperature Polaritonic limit

In this scenario, we will examine the polaritonic case where α is a large number, e.g. $\alpha \approx 10^3$. In this case, we can apply the same simplifications used above, except that we also present any rates that multiply α. For example, $A_{01}+A_{02}+E_{10}+\alpha A_{12}\approx E_{10}+\alpha A_{12}$. Using this strategy we obtain

$$\lambda_\alpha \approx \frac{\alpha(A_{01}A_{12}+A_{02}A_{12})+E_{10}A_{02}}{E_{10}+\alpha A_{12}}\,. \tag{82}$$

This is an excellent approximation, as can be seen in Fig. S13.

10.1.3 Temperature dependence of rate ratios

Equipped with Eqs. 81 and 82, we can write an expression for the rates ratio

$$r=\frac{\lambda_\alpha}{\lambda_1}\approx \frac{\alpha(A_{01}A_{12}+A_{02}A_{12})+E_{10}A_{02}}{A_{02}(E_{10}+\alpha A_{12})}\,. \tag{83}$$

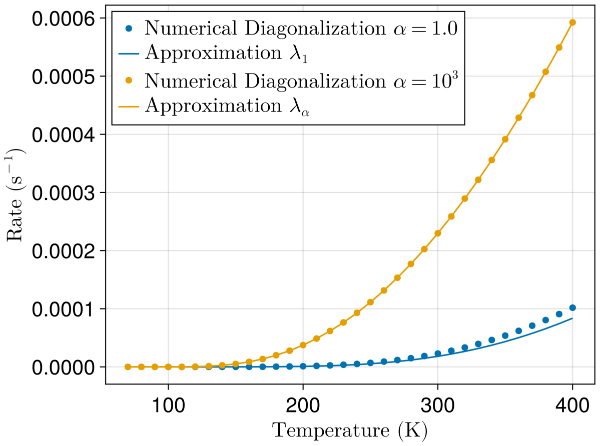

Figure S13: Comparison between BIRD rates obtained via numerical diagonalization and approximations derived in Eqs. 81 and 82.

Each rate, except for spontaneous emissions, depends on the temperature through the Bose-Einstein factor. That is,

$$A_{ij}(T) = \frac{W_{ij}}{e^{\beta\epsilon_{ij}} - 1} , \tag{84}$$

$$E_{ij}(T) = W_{ij}\left(1 + \frac{1}{e^{\beta\epsilon_{ij}} - 1}\right) , \tag{85}$$

where $\beta = \frac{1}{kT}$ and $W_{ij} = \frac{\omega_{ij}|\mu_{ij}|^2\pi}{3\epsilon_0\hbar}D(\omega_{ij})$. For our qualitative analysis, we will employ the low-temperature approximation $(e^{\beta\epsilon_{ij}} - 1)^{-1} \approx e^{-\beta\epsilon_{ij}}$. Hence,

$$A_{ij}(T) = W_{ij}e^{-\beta\epsilon_{ij}} , \tag{86}$$

$$E_{ij}(T) = W_{ij}\left(1 + e^{-\beta\epsilon_{ij}}\right) , \tag{87}$$

where the 1 in the parenthesis of Eq. 87 comes from the spontaneous emission part, which is temperature-independent. The polaritonic enhancement (i.e., the ratio of reaction rates) can be modeled as

$$r = \frac{\alpha W_{12}(W_{01}e^{-\beta\epsilon_{01}}e^{-\beta\epsilon_{12}} + W_{02}e^{-\beta\epsilon_{02}}e^{-\beta\epsilon_{12}}) + W_{02}W_{10}e^{-\beta\epsilon_{02}}(1 + e^{-\beta\epsilon_{10}})}{W_{02}e^{-\beta\epsilon_{02}}(W_{10} + W_{10}e^{-\beta\epsilon_{10}} + W_{12}\alpha e^{-\beta\epsilon_{12}})} . \tag{88}$$

Dividing every terms by $e^{-\beta\epsilon_{02}}$ and recognizing that $e^{-\beta\epsilon_{01}}e^{-\beta\epsilon_{12}}e^{\beta\epsilon_{02}} = 1$ we get

$$r = \frac{\alpha W_{01}W_{12} + W_{02}W_{10}(1+e^{-\beta\epsilon_{10}}) + W_{12}W_{02}\alpha e^{-\beta\epsilon_{12}}}{W_{02}W_{10}(1+e^{-\beta\epsilon_{10}}) + W_{12}W_{02}\alpha e^{-\beta\epsilon_{12}}} \tag{89}$$

$$= 1 + \frac{\alpha W_{01}W_{12}}{W_{02}(W_{10} + W_{10}e^{-\beta\epsilon_{10}} + \alpha W_{02}e^{-\beta\epsilon_{12}})} \tag{90}$$

For simplicity, we make the qualitative approximation $W_{01} \approx W_{12} \approx W_{02}/2$ to get the final expression

$$r \approx 1 + \frac{\alpha}{2[1+e^{-\beta\epsilon}(1+2\alpha)]}\ , \tag{91}$$

with $\epsilon = \frac{1}{2}(\epsilon_{01}+\epsilon_{12})$. As $T \to 0$, r approaches $1+\alpha/2$. For large a and large T, the expression approaches a constant value of $r = 1+\frac{1}{4}$. Note, however, that this is not a good approximation for the ratio at high-temperatures, since Eq. 91 was derived for low temperatures where spontaneous emission is the dominant process. Nevertheless, this Eq. 91 gives us a qualitative picture of how polaritonic enhancement changes when temperatures are decreased. This behavior is illustrated in Fig. S14 where we verify that this expression sucessfully captures the qualititive temperature dependence of the reaction ratios.

The model derived here predicts that polaritonic enhancement is maximized at low temperatures. At high enough temperatures or small enough values of α (such as in the weak coupling regime), Eq. 91 will be fairly constant and rate ratios will be perceived as temperature independent. While the proper bounds of polariton enhancement will depend on the exact energetics and transition dipole moments of the system, we expect that the temperature dependence to hold. In fact, a similar trend where r increases rapidly at low enough temperatures is also observed for the LiH, HF, and NaF molecules.

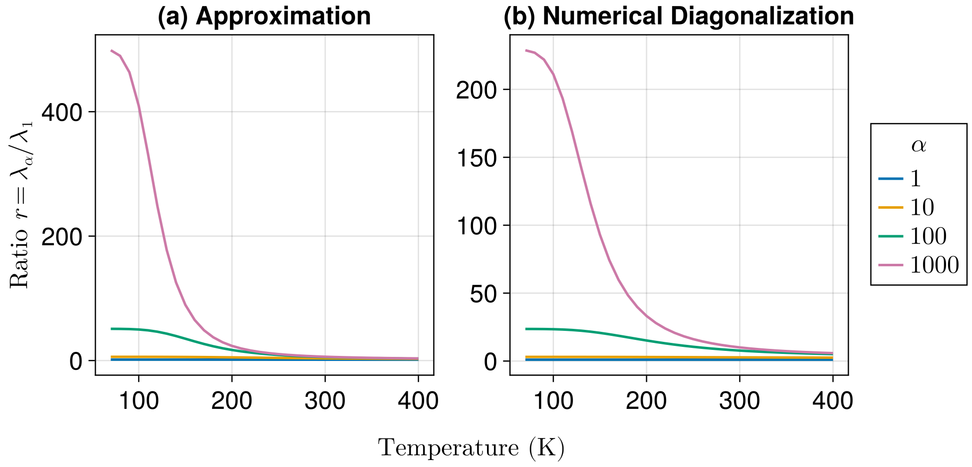

Figure S14: Polariton enhacement factors measured by the ration between rates with $\alpha \gg 1$ and $\alpha = 1$ computing using (a) approximate expression given in Eq. 91 and (b) numerical diagonalization of the transport matrix described in Eq. 73

11 Rates without Overtones

The sensitivity analysis in Sec. 4 revealed that specific overtone transitions act as bottlenecks for the investigated diatomic BIRD from the most energetic bound state. In this section, we provide computations in the weak coupling regime that include only transitions between nearest energy levels $i \to i \pm 1$ and ignore the effects of overtones. The results are shown in Fig. S15 and Table S6, which show that overtones are essential for accurately capturing the BIRD kinetics inside and outside a microcavity.

Fig. S15 shows relative rates for a range of microcavity lengths in the weak coupling regime. Note that Fig. S15 is similar to Fig. 2 of the main text, but only transitions between neighboring levels are allowed here. The results are qualitatively different. The complicated pattern seen in Fig. 2 is substituted by a much simpler function that can be fully understood by analyzing a single transition. A sensitivity analysis reveals that the most important transitions for HF and LiH are $22 \to 23$ and $28 \to 29$, respectively. These transitions correspond to $i_{\text{max}} - 1 \to i_{\text{max}}$ and have very small frequencies, 149.8 for HF and 61.3 cm^{-1} for LiH. Thus, oscillations in the relative rates (k_c/k_0) with changes in microcavity length occur over much larger periods (compare the axes of Fig. 2 and Fig. S15). Dotted green and pink lines in Fig. S15 represent the relative microcavity DOS at 149.8 and 61.3 cm^{-1}, demonstrating the effect on the BIRD rate depends only on the photon DOS at the $i_{\text{max}} - 1 \to i_{\text{max}}$ transition frequency. Finally, we also emphasize the quantitative effect of neglecting overtones. The free space rates (k_0) are reduced by three orders of magnitude when the model includes only fundamental transitions (Table S6).

Table S6: Computed free space BIRD rates (k_0) with and without overtone transitions at $T = 2000$ and 4000 K for LiH and HF, respectively.

	k_0 without overtones (s^{-1})	k_0 with overtones (s^{-1})
HF	5.68×10^{-9}	2.70×10^{-6}
LiH	7.73×10^{-8}	1.64×10^{-5}

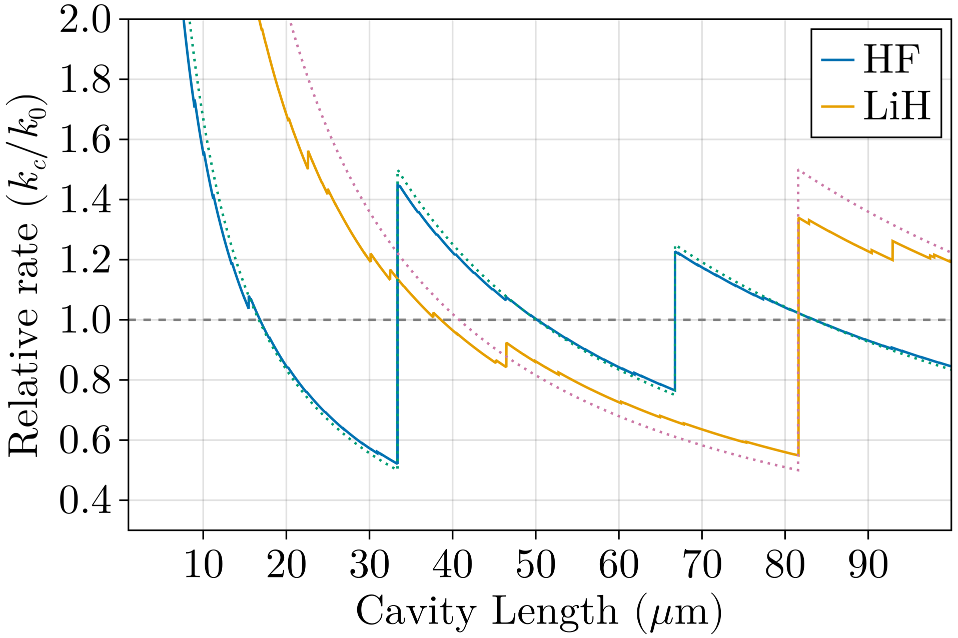

Figure S15: Ratio of BIRD rate, computed without overtones, inside a microcavity (weak coupling regime) to the free-space rate as a function of microcavity length for HF and LiH molecules. Dotted green and pink lines represent the relative density of states, $D_C(\omega)/D_0(\omega)$, at $\omega = 149.8$ cm^{-1} and $\omega = 61.3$ cm^{-1}, respectively.

References

(1) Kaluža, M.; Muckerman, J. T.; Gross, P.; Rabitz, H. Optimally controlled five-laser infrared multiphoton dissociation of HF. *The Journal of Chemical Physics* **1994**, *100*, 4211–4228.

(2) Fedorov, D. A.; Derevianko, A.; Varganov, S. A. Accurate potential energy, dipole moment curves, and lifetimes of vibrational states of heteronuclear alkali dimers. *J. Chem. Phys.* **2014**, *140*.

(3) Stine, J.; Noid, D. Classical treatment of the dissociation of hydrogen fluoride with one and two infrared lasers. *Opt. Commun* **1979**, *31*, 161–164.

(4) Guldberg, A.; Billing, G. D. Laser-induced dissociation of hydrogen fluoride. *Chem. Phys. Lett* **1991**, *186*, 229–237.

(5) Gross, P.; Neuhauser, D.; Rabitz, H. Teaching lasers to control molecules in the presence of laboratory field uncertainty and measurement imprecision. *J. Chem. Phys.* **1993**, *98*, 4557–4566.

(6) Irikura, K. K. Experimental Vibrational Zero-Point Energies: Diatomic Molecules. *JPCRD* **2007**, *36*, 389–397.

(7) Stwalley, W. C.; Zemke, W. T. Spectroscopy and Structure of the Lithium Hydride Diatomic Molecules and Ions. *JPCRD* **1993**, *22*, 87–112.

(8) Dulick, M.; Zhang, K.-Q.; Guo, B.; Bernath, P. Far- and Mid-Infrared Emission Spectroscopy of LiH and LiD. *J. Mol. Spectrosc.* **1998**, *188*, 14–26.

(9) Nasiri, S.; Shomenov, T.; Bubin, S.; Adamowicz, L. Dissociation energy and the lowest vibrational transition in LiH without assuming the non-Born–Oppenheimer approximation. *Mol. Phys.* **2022**, *120*.

(10) Holka, F.; Szalay, P. G.; Fremont, J.; Rey, M.; Peterson, K. A.; Tyuterev, V. G. Accurate ab initio determination of the adiabatic potential energy function and the Born–Oppenheimer breakdown corrections for the electronic ground state of LiH isotopologues. *J. Chem. Phys.* **2011**, *134*.

(11) Tung, W.-C.; Pavanello, M.; Adamowicz, L. Very accurate potential energy curve of the LiH molecule. *J. Chem. Phys.* **2011**, *134*.

(12) Dahl, J. P.; Springborg, M. The Morse oscillator in position space, momentum space, and phase space. *J. Chem. Phys.* **1988**, *88*, 4535–4547.

(13) Dunbar, R. C. BIRD (blackbody infrared radiative dissociation): evolution, principles, and applications. *Mass spectrometry reviews* **2004**, *23*, 127–158.

(14) Valance, W. G.; Schlag, E. W. Theoretical Rate Constant for Thermal Unimolecular Reactions in a Multilevel System. *The Journal of Chemical Physics* **1966**, *45*, 216–223.

(15) Gilbert, R. G.; Smith, S. C. *Theory of Unimolecular and Recombination Reactions*; Blackwell Scientific Publications: Oxford, UK, 1990.

(16) Hänggi, P.; Talkner, P.; Borkovec, M. Reaction-rate theory: fifty years after Kramers. *Rev. Mod. Phys.* **1990**, *62*, 251–341.

(17) Pillai, M.; Goglio, J.; Walker, T. G. Matrix Numerov method for solving Schrödinger's equation. *American Journal of Physics* **2012**, *80*, 1017–1019.

(18) Barnes, W. L.; Horsley, S. A. R.; Vos, W. L. Classical antennas, quantum emitters, and densities of optical states. *J. Opt.* **2020**, *22*, 073501.

(19) Zoubi, H.; La Rocca, G. Microscopic theory of anisotropic organic cavity exciton polaritons. *Physical Review B* **2005**, *71*, 235316.

(20) Jackson, J. D. *Classical electrodynamics*; John Wiley & Sons, 2021.

(21) Ashida, Y.; Imada, M.; Ueda, M. Cavity Quantum Electrodynamics at Strong Coupling: Polariton Physics in Nonequilibrium Open Systems. *Review of Modern Physics* **2021**, *93*, 025005.

(22) Craig, D.; Thirunamachandran, T. *Molecular Quantum Electrodynamics: An Introduction to Radiation-molecule Interactions*; Dover Books on Chemistry Series; Dover Publications, 1998.

(23) Power, E. A.; Thirunamachandran, T. Quantum Electrodynamics in a Cavity. *Physical Review A* **1982**, *25*, 2473–2484.

(24) Todorov, Y.; Sirtori, C. Intersubband Polaritons in the Electrical Dipole Gauge. *Physical Review B* **2012**, *85*, 045304.

(25) Kavokin, A. V.; Baumberg, J. J.; Malpuech, G.; Laussy, F. P. *Microcavities*; Oxford University Press, 2017; Vol. 21.

(26) Yun, K. H.; Kim, D.-m.; Lee, B. J. Maximum plasmon thermal conductivity of a thin metal film. *Phys. Rev. B* **2024**, *109*, 165415.

(27) Vinogradov, E. Vibrational Polaritons in Semiconductor Films on Metal Surfaces. *Physics Reports* **1992**, *217*, 159–223.

(28) Ashida, Y.; İmamoğlu, A. m. c.; Faist, J.; Jaksch, D.; Cavalleri, A.; Demler, E. Quantum Electrodynamic Control of Matter: Cavity-Enhanced Ferroelectric Phase Transition. *Phys. Rev. X* **2020**, *10*, 041027.

(29) Born, M.; Wolf, E. *Principles of optics: electromagnetic theory of propagation, interference and diffraction of light*; Elsevier, 2013.
