# Supplementary material for: Polaritonic Control of Blackbody Infrared Radiative Dissociation
Source: J Phys Chem Lett. 2025 Jul 18;16(30):7530–9. doi: 10.1021/acs.jpclett.5c01475 (PMC12319914; doi:10.1021/acs.jpclett.5c01475)
Supplement: Supplementary file 2 [file jz5c01475_si_002.pdf]

jz-2025-01475y.R1

Name: Peer Review Information for "Polaritonic control of blackbody infrared radiative dissociation"

First Round of Reviewer Comments

Reviewer: 1

Comments to the Author

Suyabatmaz et. al. investigate how dissociation of a diatomic molecule is induced solely via its coupling to vacuum radiation (I use *vacuum* to mean no external sources of radiation are involved here). This process, named blackbody infrared radiative dissociation (BIRD), is only relevant at low-pressure (low-density) gases in the absence of solvent interactions. The authors investigate how coupling to various forms of radiation, such as free space radiation and confined radiation, affects the dissociation process. The authors use a simple rate theory, which in my view is a simplified secular Redfield equation, to find cavity enhancements in chemical rates.

Specifically, the authors consider three scenarios: one outside the cavity where a single diatomic molecule is coupled to the vacuum radiation of the free space, a molecule coupled to confined radiation between two reflective mirrors, and a molecule coupled to the polaritonic modes inside an optical cavity (such that the confined radiation is also coupled to material, filling the optical cavity). The authors find cavity enhances/suppresses chemical rates, with enhancement more common than suppression.

Overall, while I find the work intriguing, the authors should address my comments/criticisms before I could make a recommendation:

1. Prior works, including Ref. 16, Ref. 14, [*Nat Commun* 14, 2733 (2023), Commun. Mater. 5, 110 (2024), and Nanoph. 13, 2601-2615 (2024)] have considered cavity modification in the low solvent interaction limit (when the solvent couplings are negligibly small: energy diffusion-limited regime). Some of these works also developed analytical rate theory based on Fermi's golden rule (which should be similar to the secular redfield). Could the authors clarify if the results obtained in this work, especially when placing the molecule inside the cavity, have significant qualitative differences? Visually, the rate modifications look similar.

2. It's not clear if the present work is also limited to single-molecule strong coupling (as in these past works). The main issue with the present theoretical understanding (or the lack of) is the collective nature of the effect, wherein the cavity modification is observed when per-molecule coupling is low despite the collective coupling being sizable. I would appreciate if the authors could clarify if their present theory produces negligible cavity modifications to the chemical reactivity when coupling an ensemble of dissociating molecules.

3. I find the scenario with a dissociating molecule coupling to the polaritonic modes (in a filled cavity) a bit awkward. The premise of the work is that the BIRD rates are important at low-density gases. The cavity filled with molecules that interact with the radiation but don't have **direct** coupling to the molecule doesn't seem to resemble a real experimental scenario. I think the authors should emphasize this point when introducing the third scenario, as well as in the intro/conclusions.

4. Redfield theory only works in the weak solvent-bath coupling regime. The authors use such a crude master equation approach to capture the strong light-matter interactions. This likely overestimates the cavity modification of chemical rate. The authors (if they agree) should comment on this limitation in their paper.

Reviewer: 2

Comments to the Author

The manuscript by Suyabatmaz et al. introduces a theoretical analysis of radiative thermal dissociation of a gas-phase (diatomic) molecule in micrometer-sized microcavities, with

the idea of providing new insights into how blackbody IR radiative dissociation (BIRD) should occur differently to what is well-known in free space. The dissociation process is assumed to occur incoherently, as in free space, with Fermi-Golden rule bound-to-bound and bound-to-continuum transitions rates that are weighted by the density of final states, which is proportional to the intracavity photon density of states. As known, tuning the cavity length modifies the DOS in weak coupling. In strong coupling with an inert dielectric medium, the photon DOS is spectrally shaped by redistribution of the photon content due to Rabi splitting.

The aim is interesting and novel and the idea could have good impact in the design of next-generation intracavity chemistry experiments, but unfortunately there is an issue with the assumptions that unfortunately affect the results and probably the conclusions. It is likely that the extreme sensitivity of the relative rate in figs 2 and 3 to small sub-wavelength variations (~500 nm) of the cavity length, which is discussed extensively in terms of sharp variations of the photonic DOS at particular overtone frequencies, could be entirely an artifact of the simplified infinite quality factor microcavity model that is adopted. A much more realistic DOS model based on the Green dyadic for planar microcavities (see for instance https://doi.org/10.1103/PhysRevB.64.193308 and citing articles) that takes into account the finite reflectivity and absorptivity of the cavity mirrors (thus finite mode linewidth and no DOS singularities), would be a much better approach to treat the direct bound-to-bound ladder climbing process. The sharp oscillations around k/k0 = 1 that the authors find in Section B would likely disappear (would variations from unity still survive?). In a way this is already what they find when the bound-to-continuum mechanism is discussed in Section C: the sharp oscillations go away and net changes from free space become less pronounced.

Other model simplifications (diatomic, gas-phase, no collisions, etc) are probably not as critical to the conclusions than the oversimplified treatment of the photonic DOS, since this is precisely what determines (even qualitatively) the way in which radiative energy transfer processes occur. The authors should address this point adequately.

Author's Response to Peer Review Comments:

Response to Referees

We thank both referees for their insightful comments, suggestions, and pertinent questions. Our response to each item is given below in blue, and changes to the main manuscript are provided in *italics*.

Reviewer 1.

(1) Prior works, including Ref. 16, Ref. 14, [Nat Commun 14, 2733 (2023), Commun. Mater. 5, 110 (2024), and Nanoph. 13, 2601-2615 (2024)] have considered cavity modification in the low solvent interaction limit (when the solvent couplings are negligibly small: energy diffusion-limited regime). Some of these works also developed analytical rate theory based on Fermi's golden rule (which should be similar to the secular redfield). Could the authors clarify if the results obtained in this work, especially when placing the molecule inside the cavity, have significant qualitative differences? Visually, the rate modifications look similar.

The present work indeed connects to the earlier studies referenced. In the language used by those works, our model corresponds to the energy-diffusion-limited regime (i.e., low friction), where the reaction rate is limited by the ability of the system to accumulate sufficient energy to reach the dissociation threshold. In our framework, thermal radiation acts as a bosonic bath that provides this energy, and modifications to the photonic density of states—enabled by the cavity under both weak and strong coupling—effectively enhance this energy transfer and thus increase the reaction rate.

Four important qualitative differences distinguish our work from previous models. First, while the aforementioned prior studies adopted a double-well potential allowing for recrossing events, we use a Morse potential and assume irreversible dissociation (as is appropriate for the description of a diatomic dissociation, in contrast to double wells which would be suitable for modeling rearrangement processes). In any case, our choice to treat a gas-phase diatomic molecule dissociation process eliminates recrossing pathways and precludes the existence of a Kramers' turnover point in our model.

Second, in contrast to the previous studies where the reactive molecule forms polaritons, our model treats polaritons as passive elements that modify the radiation field. The reactive molecule itself remains weakly coupled and does not participate in polariton formation. This point is further discussed in our response to item (2).

Third, our model goes significantly beyond previous studies in treating the light-matter interaction of every vibrational quantum transition of a molecular system with resonant electromagnetic (either microcavity or polariton) modes thus providing a global treatment of the light-matter interaction and its effects on the chemical kinetics of a realistic molecular system including complete account of mechanical and electrical dipole anharmonicity, in addition to including a complete microscopic description of a perfectly reflective microcavity. In the revised version we also included a discussion of results obtained with a lossy cavity (with details in the new SI Section 8).

Last, but not least, our model distinguishes itself from the mentioned and other earlier studies in that it considers a reactive process that may proceed via multiple pathways (corresponding to different

sequences of internal vibrational transitions and especially relying on overtones), and as such the effects of blocking or activating a particular channel via enhanced friction or thermal fluctuations does not have as significant an effect as in previous studies, where the effects of confined radiation are considered only on a specific pathway, that can then be blocked or enhanced to lead to more dramatic effects on reactivity. In that sense, our description is closer to the situation with polyatomics, where blocking of a particular activation channel is unlikely to be as consequential as in a prototype molecular model where a single pathway determines the reaction effect.

To clarify these distinctions, we have added a brief comparative discussion to the introduction (page 1). The added text is provided below.

"The primary aim of this work is to characterize thermal radiative dissociation in microcavities. Whereas prior studies employed the hierarchical equations of motion and analytical theories based on Fermi's golden rule to explore similar energy-diffusion-limited reactivity modeled by double-well potentials [14,16,17,32–34], here we employ a Pauli master equation to investigate irreversible bond breaking with a Morse potential in a scenario where the microcavity or polaritons act solely as passive modifiers of the electromagnetic environment. Our methodology further differs by its microscopically detailed treatment of mechanical and electrical anharmonicity inherent to reactivity processes, and by its inclusion of the coupling of both fundamental and overtone vibrational transitions to the modified photon density of states, thereby providing a detailed description of the microcavity effect on the multiple reactive pathways available in BIRD."

(2) It's not clear if the present work is also limited to single-molecule strong coupling (as in these past works). The main issue with the present theoretical understanding (or the lack of) is the collective nature of the effect, wherein the cavity modification is observed when per-molecule coupling is low despite the collective coupling being sizable. I would appreciate if the authors could clarify if their present theory produces negligible cavity modifications to the chemical reactivity when coupling an ensemble of dissociating molecules.

We agree with the reviewer that one of the main open questions in polariton chemistry is understanding how collective light-matter interactions give rise to measurable modifications in chemical reactivity, especially when the single-molecule coupling strength is weak. In our model, the dissociating molecule remains weakly coupled to the electromagnetic field and does not participate in polariton formation. In our first scenario, the diatomic molecule is coupled to an empty microcavity, whereas in the second analyzed situation, the diatomic is weakly coupled to a microcavity that is strongly coupled with a host material with large oscillator strength, which fills the cavity and modifies the photonic environment.

We did not consider the case where the reactive diatomic is member of an ensemble that couples collectively to the microcavity, as this is a topic we intend to pursue in future work with other techniques. New issues arise in this case as multiple transitions of the same diatomic molecule could be in strong coupling with a multimode microcavity depending on the population of the various vibrational levels, and a detailed treatment of dephasing would be required to consider polariton decay into

reservoir modes of the reactive molecule. We respectfully prefer not to speculate on the results of future computations here.

We reiterate *this* work is focused on polaritons as modifiers of the electromagnetic density of states, rather than as hybrid excitations involving the reactive molecule. In this way, we show rate enhancements without requiring unrealistically large per-molecule coupling strengths. To emphasize this important point, we modified our methods section to include this information (page 4):

"We emphasize that the polariton modes arise from collective strong coupling between the cavity and a host material with substantial infrared oscillator strength. The reactive molecule experiences modified radiative dynamics due to changes in the photon density of states induced by the formation of polaritons, as illustrated in Figs. 1(d) and (e). Consequently, in this study, the interaction strength between our reactive diatomic and the radiation field is obtained from first principles without any scaling parameters."

(3) I find the scenario with a dissociating molecule coupling to the polaritonic modes (in a filled cavity) a bit awkward. The premise of the work is that the BIRD rates are important at low-density gases. The cavity filled with molecules that interact with the radiation but don't have direct coupling to the molecule doesn't seem to resemble a real experimental scenario. I think the authors should emphasize this point when introducing the third scenario, as well as in the intro/conclusions.

Preventing collisions is essential for observing BIRD experimentally, and some strategies to achieve that are mentioned in the manuscript (pages 2 and 4). In our third scenario, the microcavity must be either completely or partially filled with a host material to achieve collective strong coupling and generate polaritons. To study BIRD in this configuration, we assume that the reactive molecules are confined in a way that prevents direct collisions and energy exchange with the polariton-forming host. This assumption is necessary to isolate the effect of a modified photonic environment on the radiative dissociation process. We acknowledge that this setup poses experimental challenges.

Ultimately, our goal is to assess how modifications to the photonic density of states, arising from collective strong coupling, can impact the infrared radiative dissociation mechanism in isolation. This theoretical approach allows us to draw mechanistic insights without invoking unphysically large single-molecule coupling strengths or vanishing cavity volumes.

Furthermore, we know the effect of lifting the assumption of an isolated diatomic molecule in a polaritonic material. Without imposing any constraints, collisions would occur between the diatomic molecule and the host material and introduce competing nonrative vibrational energy transfer pathways that would likely further reduce the observed polaritonic effect on the dissociation rate. As such, we hypothesize our results are "best-case scenarios" or upper bounds to the polariton effect on BIRD.

The latter point was already emphasized in our original submission, yet to provide further clarification, we have followed the suggestion provided by the reviewer and added new text to the Methods (page 2) and Conclusions (page 9) sections, as described below.

In the methods section we have added:

"While this polariton-assisted scenario poses challenges for experimental realization as BIRD generally requires low-density, collision-free conditions, it is introduced here as a theoretical construct that isolates the effect of polariton-modified photonic environments on molecular infrared radiative dissociation. Nonetheless, one could envision suppressing collisions by confining reactive molecules using transparent partitions or external fields, while maintaining exposure to the confined electromagnetic modes[41-43]*. A detailed treatment of these experimental considerations, as well as the role of direct interactions between host and reactive molecules, lies beyond the scope of the present work and warrants future investigation.*

And, finally, in the conclusion:

"While our analysis ignores collisional energy transfer, and therefore may be viewed as providing upper bounds to the microcavity effect on diatomic BIRD rates, the main qualitative conclusions expressed above are expected to apply broadly to systems where thermal radiation acts as a relevant energy source.

(4) Redfield theory only works in the weak solvent-bath coupling regime. The authors use such a crude master equation approach to capture the strong light-matter interactions. This likely overestimates the cavity modification of chemical rate. The authors (if they agree) should comment on this limitation in their paper.

We thank the reviewer for raising this concern. We are happy to provide clarification below.

Our model employs a *Pauli* master equation [W. Pauli, *Festschrift zum 60. Geburtstage A. Sommerfeld* Hirzel, Leipzig, 1928 , p. 30; see also N.G. van Kampen, Physica, 1954, 603 "Quantum Statistics of Irreversible Processes" and van Hove, Physica, 1955, 517, "Quantum-mechanical perturbations giving rise to a statistical transport equation"] with fundamental and overtone transition rates derived from Fermi's golden rule to describe the population dynamics of vibrational levels in reactive diatomic molecules. As discussed in the references above and in e.g., Zwanzig's or van Kampen's textbooks, Pauli master equations are appropriate when transition rates are in general much slower than dephasing processes that randomize coherences and lead to effective (coarse-grained) classical dynamics described by population kinetic equations. In other words, our Master equation is a coarse-grained representation of the quantum kinetics justified by the slowness of the relevant infrared absorption and emission events in comparison to decoherence-inducing processes (e.g., due to the thermal rotational population which couples weakly to vibrational levels via the Coriolis force).

In addition to the conceptual points raised above, we also emphasize that, as mentioned and supported with proper citations in our manuscript, the Pauli Master equation is the central technique employed in BIRD studies outside microcavities. In essentially all of the references we have provided on BIRD, the interpretation of experiments was successfully carried out using kinetic equations similar to ours. This is

again justified by the slowness of the various transitions involved in BIRD relative to decoherence introduced by weak intramolecular interactions.

Finally, note that in contrast with molecular rearrangement reactions, recrossing events are highly unlikely for unimolecular dissociation in the assumed dilute conditions favorable for BIRD that we focused on in our study.

In view of these points, we respectfully disagree with the reviewer that our adopted master equation treatment is the cause of potential overestimate of the computed dissociation rates. In our conclusions, we recognize our estimates are only upper bounds, but this is mainly due to our neglect of non-radiative energy exchange processes which may be challenging to achieve experimentally in the polaritonic case (see our response to the previous point).

To provide further clarification on our methodology, we have added the following statements to the main manuscript on the rationale underlying our Master equation formalism (page 2):

Our analysis of BIRD in microcavities employs the same kinetic framework that has proven successful for BIRD studies in free space [24,30,35,36]. Specifically, we propagate the populations of bound vibrational levels with a Pauli master equation[37] including transition rates obtained from Fermi's golden rule[38]. This choice is justified because the infrared absorption and emission events of interest (> 100 ns) are orders of magnitude slower than rovibrational decoherence, which randomizes vibrational coherences within on a much faster timescale. The resulting separation of time scales renders the diatomic reduced density matrix effectively diagonal (in the Morse potential Hamiltonian eigenstate basis) well before any population transfer occurs, allowing the Liouville–von Neumann equation to be coarse-grained to the Pauli form[37–39-40].

We thank the reviewer again for providing insightful questions and hope the answers above and additions to the manuscript satisfactorily address all posed issues.

Reviewer 2.

Reviewer's Comments:

The manuscript by Suyabatmaz et al. introduces a theoretical analysis of radiative thermal dissociation of a gas-phase (diatomic) molecule in micrometer-sized microcavities, with the idea of providing new insights into how blackbody IR radiative dissociation (BIRD) should occur differently to what is well-known in free space. The dissociation process is assumed to occur incoherently, as in free space, with Fermi-Golden rule bound-to-bound and bound-to-continuum transitions rates that are weighted by the density of final states, which is proportional to the intracavity photon density of states. As known, tuning the cavity length modifies the DOS in weak coupling. In strong coupling with an inert dielectric medium, the photon DOS is spectrally shaped by redistribution of the photon content due to Rabi splitting.

The aim is interesting and novel, and the idea could have good impact in the design of next-generation

intracavity chemistry experiments, but unfortunately there is an issue with the assumptions that unfortunately affect the results and probably the conclusions. It is likely that the extreme sensitivity of the relative rate in figs 2 and 3 to small sub-wavelength variations (~500 nm) of the cavity length, which is discussed extensively in terms of sharp variations of the photonic DOS at particular overtone frequencies, could be entirely an artifact of the simplified infinite quality factor microcavity model that is adopted. A much more realistic DOS model based on the Green dyadic for planar microcavities (see for instance https://doi.org/10.1103/PhysRevB.64.193308 that takes into account the finite reflectivity and absorptivity of the cavity mirrors (thus finite mode linewidth and no DOS singularities), would be a much better approach to treat the direct bound-to-bound ladder climbing process. The sharp oscillations around k/k0 = 1 that the authors find in Section B would likely disappear (would variations from unity still survive?). In a way this is already what they find when the bound-to-continuum mechanism is discussed in Section C: the sharp oscillations go away, and net changes from free space become less pronounced.

Other model simplifications (diatomic, gas-phase, no collisions, etc) are probably not as critical to the conclusions than the oversimplified treatment of the photonic DOS, since this is precisely what determines (even qualitatively) the way in which radiative energy transfer processes occur. The authors should address this point adequately.

We thank the reviewer for their insightful comments and thoughtful review of our manuscript. In response, we have revised several sections of the manuscript and added a new Supporting Information Section 8, "Dissociation Rates in Lossy Cavities," which presents updated BIRD rate calculations based on dyadic Green-function methods and complex dielectric functions for Au and Al mirrors.

In the methods section, we added:

"This work focuses on establishing an upper limit for microcavity effects on BIRD by assuming perfectly reflecting mirrors. Real microcavities are leaky, so to assess the robustness of our predictions, we also computed the photon density of states and corresponding quantum state transition rates in imperfect microcavities with finite reflectivity and absorbing metallic mirrors using the electromagnetic field dyadic Green function[55] (see Supplementary Information for details)."

As indicated by the above statements, we performed additional simulations using a Green's function based approach to compute the electromagnetic DOS of lossy planar cavities with realistic metal mirrors (Au, Al, Pt), as described in detail in the new Supporting Information Section 8. These results incorporate the finite transmissivity and absorptivity of metallic mirrors via Drude-model dielectric functions and demonstrate that, in fact, the sharp oscillations in the relative dissociation rate of a diatomic in a nearly empty microcavity observed under variation of the cavity length are indeed suppressed when mirror losses are included. The resulting DOS and dissociation rates of lossy metallic microcavities are smoother (Fig. S8 and S9, respectively), yet they still exhibit structured deviations from the free-space limit, especially at short cavity lengths.

Interestingly, we also find that lossy microcavities with metallic mirrors can exhibit DOS and rate enhancements relative to the microcavity with perfect mirrors due to the contributions from evanescent fields near the metal interfaces which only arise in leaky cavities. These near-field modes increase the

1515 Dickey Drive, Emory University, Atlanta, Georgia, 30322
Phone: (404) 727-CHEM Website: www.chemistry.emory.edu

local electromagnetic energy density accessible to molecular transitions especially at short microcavity lengths and lead to the enhancements clearly observed at low frequencies (Figs. S8) and reduced cavity lengths (Fig. S9).

Nevertheless, while the additional effects of leaky mirrors are intriguing, the order of magnitude of dissociation rate enhancements and weak suppression induced by the microcavity remains unchanged relative to the case with perfect mirrors (Fig. S9). Therefore, for brevity, we placed most of the details related to the dissociation rate computations in lossy microcavities in the new Supplementary Information section and added the following comments to the discussion of BIRD in an empty microcavity (Section IIIA, page 6):

We conclude this section by noting that BIRD simulations with lossy mirrors (SI Sec. 8) show (i) broadening and suppression of the sharp features in Fig. 2 and (ii) enhanced dissociation rates relative to Fig. 2(a) at short L_C due to the prescence of evanescent modes with significant amplitude near the imperfect metal interfaces[7,61]. Overall, lossy mirrors suppress the oscillatory behavior of the relative BIRD rate with microcavity length while preserving the order-of-magnitude effects predicted for perfect mirrors. This confirms that, modulo the fast oscillations in Fig. 2(a), the results presented in this Section are representative of experimentally accessible photon resonators

The aforementioned treatment of the impact of lossy mirrors was applied specifically to the empty microcavity case described in Section 3A of our manuscript. Yet, these findings are highly suggestive that realistic metallic mirrors would influence the polariton-assisted rates in the same qualitative way: sharp spectral features will become smoother, yet the order-of-magnitude of BIRD enhancements predicted for microcavities with perfect mirrors are very likely to persist (as they did in the previously analyzed and explicitly computed weak coupling scenario).

In addition, our polariton-assisted simulations already include an effective light-matter-coupling cutoff that eliminates highly off-resonant couplings and effectively removes the polariton stop gap singularity from consideration, so that incorporating cavity loss is not expected to change our conclusions regarding the order of magnitude of the observed polariton-assisted rate enhancement. Nevertheless, we recognize that a detailed treatment of microcavity losses in BIRD is relevant and we intend to address this in future work treating polyatomic molecules and introducing intramolecular vibrational relaxation as well as nonradiative collisional energy transfer.

We added the following additional comment to our previous discussion of the expected effect of lossy mirrors and other sources of imperfections on the polariton-assisted rates (page 7):

"Still, given the lack of an impact of leaky mirrors on the order of magnitude of the microcavity effect on the BIRD rates discussed in Sec. III.A and SI Sec. 8, we expect the order of magnitude of polariton-assisted BIRD enhancement to be similarly robust to the introduction of microcavity losses characteristic of experimentally accessible devices."

We hope these new simulations and modifications to our manuscript and SI address the reviewer's concerns.

1515 Dickey Drive, Emory University, Atlanta, Georgia, 30322
Phone: (404) 727-CHEM Website: www.chemistry.emory.edu
